# Topological data analysis of zebrafish patterns

**DOI:** 10.1073/pnas.1917763117

**Published:** 2020-02-25

**Authors:** Melissa R. McGuirl, Alexandria Volkening, Björn Sandstede

**Affiliations:** ^a^Division of Applied Mathematics, Brown University, Providence, RI 02912;; ^b^NSF–Simons Center for Quantitative Biology, Northwestern University, Evanston, IL 60208;; ^c^Data Science Initiative, Brown University, Providence, RI 02912

**Keywords:** topological data analysis, agent-based model, self-organization, pattern quantification, zebrafish

## Abstract

Self-organized pattern behavior is ubiquitous throughout nature, from fish schooling to collective cell dynamics during organism development. Qualitatively these patterns display impressive consistency, yet variability inevitably exists within pattern-forming systems on both microscopic and macroscopic scales. Quantifying variability and measuring pattern features can inform the underlying agent interactions and allow for predictive analyses. Nevertheless, current methods for analyzing patterns that arise from collective behavior capture only macroscopic features or rely on either manual inspection or smoothing algorithms that lose the underlying agent-based nature of the data. Here we introduce methods based on topological data analysis and interpretable machine learning for quantifying both agent-level features and global pattern attributes on a large scale. Because the zebrafish is a model organism for skin pattern formation, we focus specifically on analyzing its skin patterns as a means of illustrating our approach. Using a recent agent-based model, we simulate thousands of wild-type and mutant zebrafish patterns and apply our methodology to better understand pattern variability in zebrafish. Our methodology is able to quantify the differential impact of stochasticity in cell interactions on wild-type and mutant patterns, and we use our methods to predict stripe and spot statistics as a function of varying cellular communication. Our work provides an approach to automatically quantifying biological patterns and analyzing agent-based dynamics so that we can now answer critical questions in pattern formation at a much larger scale.

## Significance

While pattern formation has been studied extensively using experiments and mathematical models, methods for quantifying self-organization are limited to manual inspection or global measures in many applications. Our work introduces a methodology for automatically quantifying patterns that arise due to agent interactions. We combine topological data analysis and machine learning to provide a collection of summary statistics describing patterns on both microscopic and macroscopic scales. We apply our methodology to study zebrafish patterns across thousands of model simulations, allowing us to make quantitative predictions about the types of pattern variability present in wild-type and mutant zebrafish. Our work helps address the widespread challenge of quantifying agent-based patterns and opens up possibilities for large-scale analysis of biological data and mathematical models.

**Fig. 1.**
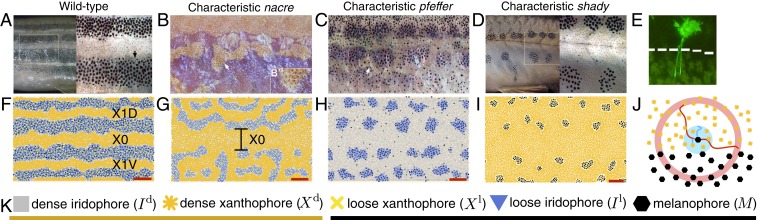
Self-organization during development. Diverse skin patterns form on zebrafish due to the interactions of pigment cells. (*A*) Wild-type zebrafish feature dark stripes and light interstripes (4, 11), while mutant patterns that form because a particular cell type is missing have altered, more variable patterns. (*B*) The *nacre* mutant (encoding mitfa) (9, 12) has an enlarged central orange region flanked by blue patches. (*C*) *Pfeffer* (encoding csf1rA) (9, 10, 13) is characterized by messy spots arranged horizontally (11). (*D*) *Shady* (encoding ltk) (11, 14) often features smooth black spots roughly arranged in stripes. Reproduced from ref. 11, which is licensed under CC BY 3.0. (*E*) Pigment cells extend long legs (measuring up to half a stripe width in distance) toward interstripe cells for communication (26). Reproduced from ref. 26, which is licensed under CC BY 3.0. (*F–I*) The agent-based model (20) replicates zebrafish patterns in silico. (Red scale bar, 500 **μ**m throughout this paper.) The central light interstripe is labeled X0, and the next two interstripes are called X1V and X1D (11). (*J*) Rules for agent behavior in the model (20) depend on the cells in short-range disks and a long-range annulus. Reproduced from ref. 20, which is licensed under CC BY 4.0. (*K*) Summary of the main pigment cells involved in patterning. Interstripes consist of orange dense xanthophores and silver dense iridophores, and stripes contain yellow loose xanthophores, blue loose iridophores, and black melanophores.

Patterns are widespread in nature and often form due to the self-organization of independent agents. Whether exploring such collective dynamics in cancer (1), wound healing (2), hair growth (3), or skin pattern formation (4, 5), researchers focus on uncovering unknown cell behavior and signaling using a combination of experimental and modeling techniques. This process is complicated by the fact that biological patterns are inherently variable, making it challenging to quantify the distinguishing features of different mutants and judge model accuracy. In some applications, such as zebrafish skin patterns (Fig. 1 *A*–*D*), global information about patterns both in vivo and in silico is largely based on visual inspection, and this naturally leads to more subjectivity and limits the scale of the analyses. Moreover, the focus is often on the characteristic features of different mutants, making it unclear how much variability normally arises in mutant patterns and how this variability compares to wild type. To help address these challenges, here we develop a methodology, based on topological data analysis and machine learning, for quantifying self-organized patterns with an automated, agent-based approach, and we apply our methods to study variability in zebrafish skin patterns.

Characterized by black and gold stripes, the zebrafish (*Danio rerio*) is a model organism in the field of skin pattern formation (4, 6, 7). Remarkably, zebrafish stripes form due to the interactions of tens of thousands of different-colored cells, which reliably self-organize on the growing skin despite their stochastic environment (8–10). In addition to their namesake stripes, zebrafish feature a wealth of other patterns [e.g., spots and labyrinth curves (11)] that form due to genetic mutations that restrict cell birth or alter cell behavior (often in unknown ways). While wild-type stripes (Fig. 1*A*) are considered robust, mutants that lack certain cell types (Fig. 1 *B*–*D*) feature more variable spotty patterns (11). For example, the *nacre* phenotype (9, 11, 12) has an enlarged central orange region with scattered blue splotches (Fig. 1*B*). In comparison, both the *pfeffer* (9–11, 13) and *shady* (11, 14) mutants are characterized by dark spots, roughly aligned in stripes. These patterns differ in their finer details: *pfeffer* has messy spots and peppered black cells across its skin, while *shady* has sharp boundaries between light and dark regions (11). Although these descriptions apply in general, patterns vary due to the stochastic nature of pigment cell interactions.

Mathematical descriptions of zebrafish patterns capture stochastic cellular interactions at different levels of detail. While partial differential equations (e.g., refs. 8, 15, and 16) offer a broad perspective on the evolution of cell densities, cellular automaton (17, 18) and agent-based models (19–21) provide a more detailed view of individual cell behavior. For example, the agent-based model (20) specified cell interactions using stochastic rules to simulate zebrafish patterning in silico (Fig. 1 *F*–*I*). Ideally, models should reproduce pattern formation as it is observed in vivo, and this raises the question, How can we systematically quantify and compare pattern features, particularly in the presence of biologically induced variability? Moreover, researchers seek to identify the cell interactions that are altered in mutant patterns, but this process is limited by the large number of parameters in agent-based models and the need for visual inspection to analyze simulation results. Reliable, automatic quantification of patterns (for both in vivo and in silico data) is therefore fundamental to measuring how well models perform and increasing their predictive potential.

Many black-box machine-learning algorithms have been developed for pattern classification, but these approaches require extensive training data and tedious manual labeling. Interpretable methods provide results with a more transparent relationship to biological data. In this vein, Lee et al. (22) showed how to use ImageJ (23) to quantify traits of giraffe spots; while their process can be automated, it relies on data in the form of contiguous blocks of bits in an image and captures only macroscopic pattern features, losing the underlying discrete, cell-based nature of the data. Taking a different approach, Miyazawa et al. (24) assigned a “pattern simplicity score” (associated with the circularity of black–white boundary contours) to images of salmon patterns, and they quantified overall color tone by calculating the ratio of light to dark areas on fish images. These two global measures, which were applied to trout in ref. 25, are broadly applicable but are not intended to capture detailed features. The methodology that we introduce in this paper, in contrast, utilizes the cell-based nature of skin patterns to quantify both macroscopic pattern attributes and microscopic features on the cellular level.

As shown in ref. 27, topological data analysis (TDA) has emerged as a valuable tool for characterizing collective behavior and self-organization. Tools from TDA, specifically persistent homology, allow one to assign shape descriptors to noisy or large data across a range of spatial scales and, in contrast to deep learning, they do not rely on any labeled training data. In the case of collective behavior, this translates to measuring topological summaries (e.g., connected components and loops) of the resulting patterns from the cellular level to the global level. In ref. 27, TDA was applied to study the velocity and positions of agents in simulations of a flocking model. By tracking global persistent homology features over time, Topaz et al. (27) were able to identify agent clusters and detect the presence of global dynamics that would be challenging to notice visually. While such prior work (24, 25, 27) has demonstrated how to quantify various overall features of patterns, characterizing the distinguishing traits of the different zebrafish patterns in Fig. 1 at the level of pigment cells requires a more detailed perspective.

Inspired by the utility of TDA for quantifying collective behavior, here we show how to reinterpret topological summaries as detailed measurements of pattern features. By combining TDA with interpretable machine-learning techniques and working closely with the biological literature on zebrafish, we are able to automatically detect and quantify patterns given agent (e.g., cell) coordinate data. Our main contribution is an automated, interpretable framework for counting stripes and spots, detecting broken stripes, measuring stripe widths, quantifying stripe straightness, calculating spot size and roundness, measuring spot placement, and estimating the onset of stripe formation from pattern data. To illustrate our techniques, we apply our methods to thousands of in silico images of zebrafish patterns generated using the agent-based model from ref. 20. Because zebrafish display a wide range of patterns, we expect that our methodology can be applied to other problems in biological self-organization as well as to in vivo data. Our approach opens up a range of possibilities for large-scale analysis of experimental images to better understand the cellular mechanisms underlying pattern formation.

## Background and Methods

Here we give a brief overview of zebrafish biology and the model (20), as well as an introduction to the TDA and machine-learning concepts that we use in our methods [see *SI Appendix* for additional background on TDA and the model (20), including its biological basis].

### Biological Background.

Zebrafish stripe patterns consist of three main types of pigment cells: black melanophores, yellow/orange xanthophores, and silver/blue iridophores (11) (Fig. 1*A*). Xanthophores and iridophores are spread across the skin in two forms (dense in light interstripes and loose in dark stripes), while black cells reside only in stripes (10, 28–30). As these cells undergo differentiation, division, death, migration, and form changes, they self-organize into four to five stripes and four interstripes sequentially over a few months (4). During this time, the fish body grows in length from roughly 7.5 mm to over 16 mm (31). Cells regulate each other’s behavior through communication at short range (between neighboring cells) and at long range (between cells in stripes and interstripes) (e.g., refs. 8, 15, and 32–34); see Fig. 1*E*. Importantly, this regulation is inherently noisy. For example, cells may interact by reaching extensions toward their neighbors (26, 35, 36); whether or not cellular communication occurs then depends on whether these extensions successfully find another cell.

Prior models (19, 20) have used estimates of wild-type stripe width (26, 37) and descriptions of developmental timelines (e.g., approximate times at which new stripes appear) (4, 31, 38) to judge model performance or fit parameters. Fewer data are available for zebrafish mutants, and, to our knowledge, global information is in the form of qualitative descriptions of the characteristic features of their patterns. Local measurements, in turn, include cell speeds (39, 40) and distances between adjacent cells (33, 39, 41). Notably, we are not aware of measurements of pattern variability or stripe straightness.

### Model and Generation of In Silico Pattern Data.

The model (20) treats pigment cells as individual agents (point masses) and tracks their positions (namely (x,y) coordinates) in space as they interact on growing 2D domains. These domains capture the full height of the fish body and one-third of its length (excluding a region around the eye). The number of agents is carefully based on empirical measurements of cell–cell distances [roughly 30 to 80 μm, depending on the cell type (39)], so that agent dynamics occur on the same scale as cell interactions on the fish skin (20). See *SI Appendix*, Fig. S3 for a summary of the model (20) and the length scales involved.

The behavior of five different types of cell agents is accounted for in ref. 20: We let $M_{i}\left(t\right)$ be the (x,y) coordinate of the ith melanophore (M) at time t; similarly, $X_{i}^{\text{d}}\left(t\right)$, $X_{i}^{\text{l}}\left(t\right)$, $I_{i}^{\text{d}}\left(t\right)$, and $I_{i}^{\text{l}}\left(t\right)$ denote the locations of the ith dense xanthophore ($X^{\text{d}}$), loose xanthophore ($X^{\text{l}}$), dense iridophore ($I^{\text{d}}$), and loose iridophore ($I^{\text{l}}$), respectively; see Fig. 1*K*. Space is continuous, and cell movement, which includes repulsion and attraction, is modeled by coupled ordinary differential equations. Cell birth, death, and transitions in type, in turn, take the form of stochastic, discrete-time rules. These rules, which are strongly motivated by the biological literature (e.g., refs. 11, 15, 34, and 39), depend on the number of cells in disk and annulus neighborhoods centered at the cell or location of interest (Fig. 1*J*). Volkening and Sandstede (20) use these neighborhoods to model the cells that a given cell (or precursor) could communicate with [e.g., through direct contact (42), diffusing substances (34), or dendrite extensions (26, 35, 36) as in Fig. 1*E*]. As an example cell interaction rule, interstripe cells are known to promote M differentiation at long range (15, 32), and these dynamics are modeled as

$$\begin{array}{ll} & \frac{\sum_{i=1}^{N_{\text{X}}^{\text{d}}}1_{\Omega_{\text{long}}^{z}}\left(X_{i}^{\text{d}}\right)+\sum_{i=1}^{N_{I}^{\text{d}}}1_{\Omega_{\text{long}}^{z}}\left(I_{i}^{\text{d}}\right)}{\alpha +\beta \sum_{i=1}^{N_{\text{M}}}1_{\Omega_{\text{long}}^{z}}\left(M_{i}\right)}>1\\ & \Rightarrow M\;\text{birth at}\;z\left(\text{if not overcrowded}\right), \end{array}$$ [1]

where z is a randomly selected location to be evaluated for possible cell birth; $N_{\text{X}}^{\text{d}}$, $N_{\text{I}}^{\text{d}}$, and $N_{\text{M}}$ are the numbers of $X^{\text{d}}$, $I^{\text{d}}$, and M cells on the domain, respectively; and $\Omega_{\text{long}}^{z}$ is an annulus centered at z that models long-range cellular communication (Fig. 1*J*). According to Eq. **1**, a new M cell appears at position z when the ratio of interstripes cells to M cells at long range is greater than one. [Note that the interaction rules in ref. 20 are given in terms of numbers, rather than proportions, of cells. We have adjusted the model (20) so that these rules depend on the ratios or densities of cells in different regions, as this framework works better for our large-scale study; see *SI Appendix* for more details.]

The agent-based model (20) can be used to simulate the full timeline of adult pattern formation from when it begins when the fish is roughly 21 days post fertilization (dpf). Because the model (20) is stochastic, simulating it repeatedly leads to different in silico patterns and, importantly, for our methods, cell-coordinate data. We thus generate an extensive dataset by simulating the development of thousands of zebrafish patterns. We simulate wild-type development from 21 dpf until 66 dpf, at which point zebrafish, measuring about 2.2 mm in height and 12.6 mm in body length (according to the growth rates approximated from ref. 31 in ref. 20), are expected to have three complete interstripes, two complete stripes, and some partially formed stripes near the boundaries (*SI Appendix*, Fig. S3*B*). We simulate *nacre* and *pfeffer* pattern formation until 76 dpf and *shady* development until 96 dpf by turning cell birth off for the appropriate cell types as described in ref. 20. [We note that experimentalists often use stages (31) rather than dpf to measure time; in the model (20), 66 dpf, 76 dpf, and 39 to 44 dpf correspond to the juvenile, juvenile+, and squamation onset posterior stages, respectively.] With one exception, we perform all of our analyses on the final simulated patterns at 66 dpf (for wild type), 76 dpf (for *nacre* and *pfeffer*), and 96 dpf (for *shady*). Following the approach in ref. 20, we enforce periodic boundary conditions in the horizontal direction and wall-like boundary conditions at the top and bottom of these domains (Fig. 3*A*). To help avoid quantifying partially formed stripes or spots, we remove the cells in the top and bottom 10% of the domain in postprocessing.

To generate our first dataset, we simulate wild-type, *nacre*, *pfeffer*, and *shady* patterns under the baseline conditions and parameters described in ref. 20. We then adjust the model to account for more realistic biological stochasticity in cell interactions. In particular, rather than using deterministic length scales in the cell interaction rules, each day we select these length scales randomly per cell and interaction from a normal distribution centered at the default parameter value. In our last dataset, we focus on the inner radius of $\Omega_{\text{long}}$ in Eq. **1** and explore the role of this parameter while keeping all other parameters at their default values.

### Topological Data Analysis and Machine Learning.

**Fig. 2.**
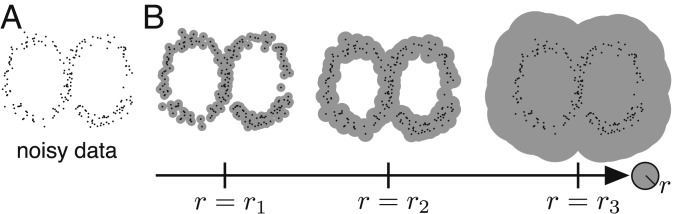
Illustration of persistent homology applied to coordinate data. (*A* and *B*) Noisy data sampled from a figure-eight shape (*A*) and corresponding manifold expansions (*B*).

Our approach to quantifying patterns relies on topological data analysis and machine learning. TDA is an emerging branch of mathematics and statistics that aims to extract quantifiable shape invariants from complex and often large data (43–47). One of the main tools in TDA is known as persistent homology, which we review now briefly. Given a dataset of N discrete points ${\left\{x_{i}\right\}}_{i=1}^{N}$ that lie in some metric space $\left(D,d_{D}\right)$, we place a ball of radius r at each $x_{i}$ to obtain the set $b_{r}\left(x_{i}\right)=\left\{y\in D:d_{D}\left(x_{i},y\right)\leq r\right\}$. We then take the union of these balls over all i∈[1,N], namely ${⋃}_{i\in \left[1,N\right]}b_{r}\left(x_{i}\right)$. This process yields a new manifold with shape generated by the original data, and persistent homology tracks how the shape of this manifold changes as r increases (Fig. 2).

For our work it suffices to view the dimension 0 and dimension 1 persistent homology groups as vector spaces whose dimensions correspond to the number of connected components and loops, respectively, of the evolving manifold (see *SI Appendix* and refs. 43–47 for more details). The number of generators of the ith homology group is called the ith betti number, denoted $\beta_{i}$. If a topological feature (e.g., connected component or loop) appears at some radius $r_{b}$ and disappears at some radius $r_{d}>r_{b}$, then we say this feature is born at $r=r_{b}$ and dies at $r=r_{d}$, and its persistence is given by $r_{d}-r_{b}$.

For example, because a figure eight has one connected component and two loops, this shape has $\beta_{0}=1$ and $\beta_{1}=2$. Now consider a noisy dataset sampled from a figure eight, as we show in Fig. 2*A*. To compute the persistent homology of these data we take the union of balls of radius r centered around each data point for an increasing sequence of r values. Two loops appear in the data at $r=r_{2}$ and disappear before $r=r_{3}$ in Fig. 2*B*, so this dataset has two dimension 1 homology generators that are both born at $r_{b}=r_{2}$ and die at $r_{d}=r_{3}$ (with persistence given by $r_{3}-r_{2}$). Similarly, this dataset is connected for $r\geq r_{2}$, so it has one dimension 0 homology generator for $r\geq r_{2}$ with infinite persistence and several dimension 0 homology generators for $r<r_{2}$. Thus, persistent homology reveals that the noisy data in Fig. 2*A* are topologically similar to a figure-eight shape ($\beta_{0}=1$, $\beta_{1}=2$) for $r_{2}\leq r<r_{3}$.

In addition to using TDA, we apply methods from interpretable machine learning to quantify patterns. Machine-learning algorithms seek to automatically learn information from a given dataset for classification or prediction purposes (48, 49). The machine-learning approach we use involves clustering data into different classes based on a similarity measure. Specifically, we apply single-linkage clustering to subsets of agents (e.g., pigment cells) to identify clusters corresponding to spot or stripe patterns. Single-linkage clustering is an agglomerative hierarchical clustering method: Each data point begins in its own cluster and points (or clusters of points) are merged sequentially based on which two clusters are closest to each other (48, 49). We continue this process until there are n clusters, where n is either one or some predetermined number of desirable clusters. We use single-linkage clustering over other clustering algorithms (e.g., average linkage or k-means) to capture elongated, undulating, and nonspherical clusters that are characteristic of some zebrafish mutants (Fig. 1).

As a side note, dimension 0 persistent homology is analogous to single-linkage clustering, so there is a natural connection between TDA- and clustering-based methods for pattern quantification (43). Using clustering and topological methods in tandem yields both multidimensional, coordinate-free summaries (from TDA) and essential information about the locations of different agents (from clustering).

## Results: Our Methodology for Quantifying Patterns

**Fig. 3.**
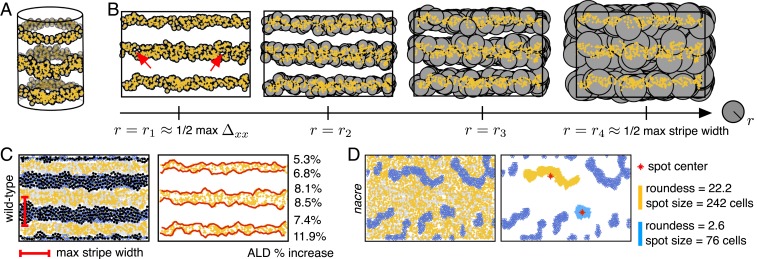
Illustration of our topological techniques applied to zebrafish patterns. (*A*) Boundary conditions are periodic in the horizontal direction, so stripes and interstripes are viewed as loops from a topological perspective. (*B*) We count interstripes and measure stripe width using persistent homology. We show manifold expansions of the locations of $X^{\text{d}}$ cells by considering balls of growing radius r centered at the location $X_{i}^{\text{d}}$ of each cell. When $r=r_{1}$, the radius of the balls is about half the maximum distance between neighboring $X^{\text{d}}$ cells $\Delta_{xx}$. At this point, three interstripes have formed, but the number of loops is larger than the true number of interstripes due to gaps between cells, highlighted by red arrows ($\beta_{0}=3$ and $\beta_{1}>3$). As r increases to $r_{2}$, the noisy loops die off, leaving only three loops ($\beta_{0}=3$ and $\beta_{1}=3$). The long persistence of three loops corresponds to the true presence of three interstripes. As r increases further to $r_{4}$, the manifold collapses to a single connected component ($\beta_{0}=1$ and $\beta_{1}=1$). The difference between the ball radius at which this collapse occurs ($r_{4}$) and the ball radius at which three loops appear ($r_{1}$) approximates half the maximum width of black stripes. (*C*) By combining TDA with clustering methods, we automatically detect interstripe boundaries and measure their curviness; we show the percentage of increase in arc length distance (ALD) of these boundaries (traced out in red) relative to perfectly straight stripes here. (*D*) We describe spotted phenotypes by combining persistent homology, clustering methods, and principal component analysis. We use $\beta_{0}$ to quantify the number of spots. As an example, we show the spot size and spot roundness for two *nacre* spots.

We now use TDA and machine learning to develop our main result: an interpretable, agent-based methodology for automatically quantifying self-organizing patterns. We summarize our methods in *SI Appendix*, Table S1 and illustrate how they can be applied to zebrafish in Fig. 3. We present direct methods for measuring local pattern features in *SI Appendix*.

Tailored to a specific application (zebrafish), our work opens up an additional way of thinking about TDA tools and using them to obtain detailed measurements of patterns. We expect that a similar approach can be used to study other patterns with data in the form of agent coordinates or images (with functional persistence). To help encourage further applications of TDA to self-organized patterns, we thus present our methods using general language in the next section, while also using zebrafish to highlight the kinds of application-specific considerations one must address when applying TDA to new data. In particular, one application-specific step involves determining what agent type(s) to use as input for topological feature computations. For example, multiple types of cells are present in the same pattern features on zebrafish (e.g., in Fig. 1*A*, both $I^{\text{d}}$ and $X^{\text{d}}$ appear in interstripes). Applying TDA to the locations of every agent type in a pattern is expensive. It may be sufficient to study only one or two agent types, but selecting which types to use requires application-specific considerations.

### Counting Spots and Stripes.

We compute the dimension 0 and dimension 1 persistent homology groups using the coordinate data of agents [e.g., pigment cell locations generated by the model (20)] to quantify pattern types, assuming periodic boundary conditions in the x direction. With these boundary conditions, spots can be viewed as connected components without loops, whereas stripes wrap around the domain and are thus connected components with a single loop (Fig. 3 *A* and *B*). Consequently, $\beta_{0}$ and $\beta_{1}$ approximate the number of spots and stripes in a pattern, respectively.*

For zebrafish, we estimate the number of stripes and interstripes in wild-type patterns by computing $\beta_{1}$ for $X^{\text{l}}$ and $X^{\text{d}}$ cells, respectively. We apply TDA to these cells because they uniformly cover the fish skin, but in different forms in stripes and interstripes.^†^ We estimate the number of spots in *nacre* and *pfeffer* patterns by computing $\beta_{0}$ using the locations of blue $I^{\text{l}}$ cells.^‡^ For *pfeffer*, individual M cells appear randomly on the domain, so using these cells to count the number of spots would introduce spurious connected components (in the form of individual black cells). In comparison, M are much more clustered in *shady*; thus, we calculate the number of dark *shady* spots by computing $\beta_{0}$ for *M*.

In general, we calculate betti numbers by applying persistent homology to the agents’ coordinates and using a persistence threshold to count the number of homological generators whose persistence is greater than the set threshold ($T_{p}$). Empirical estimates of cell–cell spacing motivate our choice of $T_{p}$ for zebrafish. Specifically, we use $T_{p}^{0}=100$ μm and $T_{p}^{0}=90\;\mu$m as the dimension 0 persistence thresholds for iridophores and melanophores, respectively. We chose these thresholds conservatively, as average xanthophore–xanthophore neighboring distances are 30 to 60 μm and average melanophore–melanophore distances are roughly 50 to 60 μm in wild type (20, 33, 39, 41). (We are not aware of empirical measurements of iridophore spacing.) For dimension 1 homology, we use a universal persistence threshold of $T_{p}^{1}=200$ μm. Moreover, to ensure that we correctly differentiate between complete and broken stripes or interstripes, we specify that a persistence generator counts toward $\beta_{1}$ only if its birth radius $r_{b}$ is below a certain threshold ($T_{b}^{1}$). For $X^{\text{l}}$ and $X^{\text{d}}$, we use $T_{b}^{1}=100\;\mu$m and $T_{b}^{1}=80\;\mu$m, respectively. These thresholds were motivated by cell–cell distance measures (33, 39, 41) and tuned based on parameter fitting experiments with stripe and interstripe breaks.

**Fig. 4.**
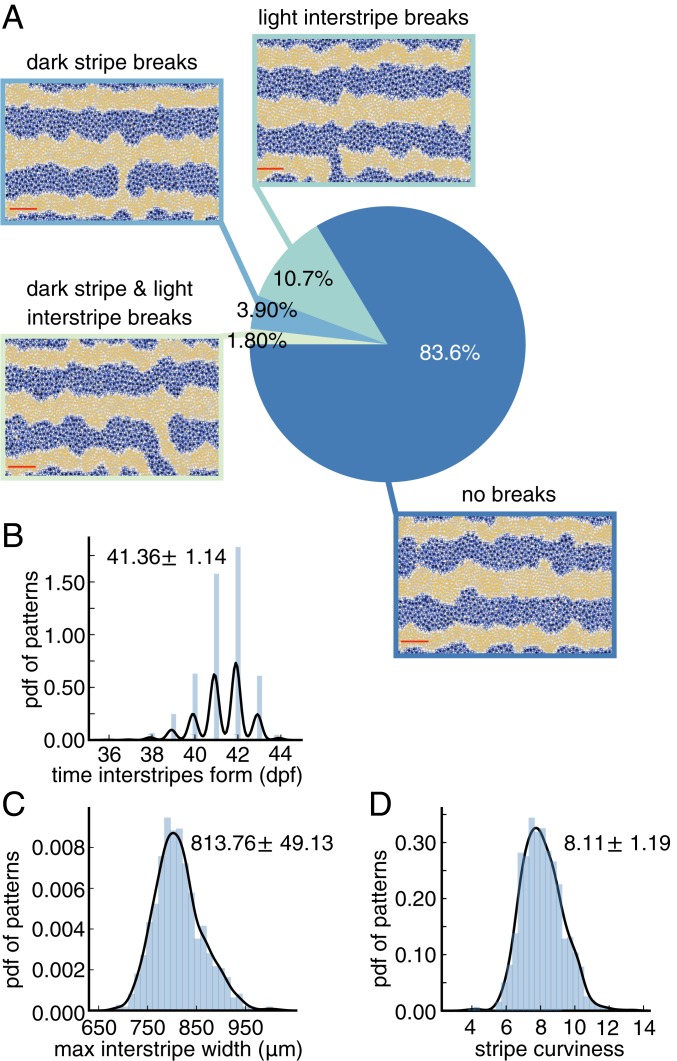
Baseline quantification of wild-type patterns. All measurements are based on 1,000 simulations of the model (20) under the default parameter regime. (*A*) We use persistent homology to detect the presence of breaks in stripes and interstripes. (Following the example in ref. 20, we do not count breaks in the dark stripes along the top and bottom boundaries of the domain.) The domain captures about one-third of the fish body (20). (*B*) Distribution of times at which interstripes X1D and X1V (Fig. 1*F*) begin to form. (*C*) Distribution of maximum interstripe width. (*D*) Distribution of stripe curviness (also see Fig. 3*C*). In *B–D*, we display histograms of in silico data and kernel density estimator (KDE) curves with a Gaussian kernel in black; the mean plus/minus the SD is shown in each plot for the data.

Simultaneously, we can use persistent homology to identify stripe breaks when the number of expected stripes is known (see Fig. 4*A* for examples of stripe and interstripe breaks). Namely, we flag a stripe break when $\beta_{1}$ is less than the expected number of stripes. Here we additionally consider $\beta_{1}$ of the M cells, with $T_{b}^{1}=90\;\mu$m and $T_{p}^{1}=200\;\mu$m. We compute $\beta_{1}$ for both $X^{\text{l}}$ and M because the former appear at low density in dark stripes; computing $\beta_{1}$ for both cell types allows us to be more confident in our results. As we discussed in *Background and Methods*, we expect that our simulated zebrafish patterns have two fully formed stripes and three fully formed interstripes at the time of our analysis, so we flag a stripe break when $\beta_{1}<2$ for both $X^{\text{l}}$ and M cells. Similarly, we flag an interstripe break when $\beta_{1}<3$ for $X^{\text{d}}$ cells.

### Measuring Stripe Width.

Beyond quantifying the number of stripes or spots, we leverage TDA to approximate stripe and interstripe widths. In particular, we estimate (inter)stripe widths using the persistence ($r_{d}-r_{b}$) of the significant dimension 1 persistence points. We define significant dimension 1 persistence points as those with persistence greater than or equal to $T_{p}^{1}$ and birth radius $r_{b}$ less than or equal to $T_{b}^{1}$. For example, the persistence of a stripe loop is the difference between the radius value ($r_{d}$) at which two adjacent stripes combine to form a single loop and the radius value ($r_{b}$) at which the stripe feature initially formed (we ignore the features that persist to infinity). This difference ($r_{d}-r_{b}$) is half of the maximum distance between two adjacent stripes, capturing the maximum width of the enclosed interstripe (Fig. 3).

In wild-type zebrafish, twice the persistence of the yellow $X^{\text{l}}$ loops yields an approximation for the maximum interstripe width across the fish. Similarly, twice the persistence of the orange $X^{\text{d}}$ loops approximates an upper bound on stripe width. We note that $r_{d}$ alone could be used as an alternative estimate for maximum (inter)stripe width, but we use $r_{d}-r_{b}$ to account for the narrow boundary region between stripes and interstripes. To obtain a lower bound on stripe width, one could calculate the persistence of the significant dimension 0 persistence points, as this measurement is based on half of the minimum distance between two adjacent interstripes.

### Measuring Spot Size.

We measure spot size by applying single-linkage hierarchical clustering to the agents of interest with the number of desired clusters (e.g., number of spots) set to the $\beta_{0}$ values we obtained from our topological analyses. Then, we count the number of cells per cluster to approximate the size of each spot. We define “spot size” as the median number of agents per spot across all of the spots.

### Quantifying Stripe Straightness.

To measure “stripe curviness” we compute the arc length distance (ALD) of the boundary of each single-linkage cluster that corresponds to a stripe. We define our stripe curviness measure to be the average percentage of increase of this ALD from the ALD of straight stripes:

$$\text{curviness}=\underset{\text{stripes}}{\text{mean}}\left(\left(\frac{\text{true ALD}}{\text{straight ALD}}-1\right)\times 100\right).$$ [2]

For example, to measure the curviness of wild-type zebrafish stripes, we apply single-linkage clustering to the locations of $X^{\text{d}}$ cells. For the number of desirable clusters n, we use the number of expected interstripes minus the number of stripe breaks that we identified with persistent homology (Fig. 3*C*). We then calculate the ALD for the resulting clusters and compute stripe curviness using Eq. **2**.

### Quantifying Spot Roundness.

To estimate spot uniformity, we use the clusters identified via single-linkage hierarchical clustering (with the number of desired clusters set to the $\beta_{0}$ values). We then apply principal component analysis (PCA) to each cluster. The eigenvalue decomposition in PCA provides information about how varied the data are in each dimension. Since our data are 2D, we use PCA to evaluate the spread of each cluster in the x and y directions. If a spot has significantly more variance in one direction, this indicates that it is irregularly shaped or elongated. Specifically, we define our roundness measure as

$$\text{roundness of spots}=\underset{\text{spots}}{\text{median}}\left(\frac{\text{PCA eigenvalue}\;1}{\text{PCA eigenvalue}\;2}\right).$$ [3]

We assume that a PCA eigenvalue ratio close to one implies round spots, while a PCA eigenvalue ratio ≫1 indicates irregular, nonuniform spots (see Fig. 3*D* for examples).

### Determining Spot Alignment and Center Width.

We quantify spot alignment by first applying single-linkage hierarchical clustering to agent locations (with the number of desired clusters set to the $\beta_{0}$ values). We then calculate the pairwise $l_{\infty }$ distances between the cluster centroids and complete a nearest-neighbor search with the $l_{\infty }$ metric.^§^ This allows us to extract the distance from each spot to its closest neighboring spot. We define the spot-spacing variance as the SD of these nearest-neighbor $l_{\infty }$ distances. A large spacing variance corresponds to nonuniform spot placement, while a small spacing variance predicts well-aligned spots.

Motivated by the *nacre* and *shady* patterns, which feature expanded light central regions (Fig. 1 *G* and *I*), we also use the cluster centroids to approximate the center width, defined as twice the distance from the midpoint of the domain to its first spot. In particular, we estimate the center width as twice the minimum distance from the cluster centroids to the midpoint of the domain, minus the median spot diameter. Here we define spot diameter as twice the greatest Euclidean distance from the spot’s centroid to cells belonging to the spot. For zebrafish, the center radius corresponds to the width of the central interstripe X0 (Fig. 1 *F* and *G*).

### Capturing Pattern Formation Events.

Thus far, we have focused on quantifying pattern features at a snapshot in time. However, for self-organization that occurs during organism development, it is also useful to estimate the time at which specific features emerge. For example, in wild-type zebrafish, the second and third interstripes X1V and X1D (Fig. 1*F*) develop around 39 to 44 dpf [based on approximations (20) of images in refs. 11 and 31]. This information on target time dynamics serves as an additional quantitative measurement that can be used to evaluate models. Here we present a method for quantifying the time at which stripes X1V and X1D form; future work could extend these methods to capture the time dynamics of spot formation and other features.

Given data in the form of agent locations at consecutive time points, we first assume new stripes form somewhere between day $d_{0}$ and $d_{1}$. If there is no prior knowledge about the expected time of stripe development, one can set $d_{0}$ and $d_{1}$ to the first and last days of pattern development, respectively. For zebrafish, because the model (20) was parameterized so that interstripes X1D and X1V form around 39 to 44 dpf, we conservatively set $d_{0}=32$ dpf and $d_{1}=62$ dpf. Within the specified time interval, we then analyze the patterns sequentially beginning at $d_{0}$, assuming there is initially a single stripe on the domain. At each time step, we find the upper and lower bounds of the stripes by computing the maximum and minimum, respectively, of the y coordinates of the agents of interest (e.g., for zebrafish, we use $X^{\text{d}}$ cells). Finally, we estimate the initial formation of new stripes as the first day at which the upper or lower bounds of the stripes increase by more than some threshold from the previous day. For zebrafish, the threshold we use is 200 μm^¶^ .

## Results: A Quantitative Study of Zebrafish Patterns

We now study zebrafish pattern variability and robustness by analyzing thousands of in silico wild-type and mutant patterns generated using the agent-based model (20). Quantitatively evaluating data of this scale is possible because of our automated framework. As a baseline test, we begin by illustrating our techniques on simulations of wild-type zebrafish stripes. Because our analysis there is consistent with previous characterizations collected visually and local pattern measurements, we then use our methods to extract quantifiable features from mutant patterns and measure pattern variability in the presence of increased stochasticity in cell interactions. We conclude by showing how our methods can be used to detect the impact of changing a given model parameter without the need for visual inspection.

We view our results in the next sections as presenting a broader, more objective picture of the behavior of the agent-based model (20). Additionally, because this model is closely based on the biological literature, our results serve to predict the kind of pattern variability we expect to see in vivo based on the model (20). As large-scale collections of experimental images become available, our predictions can be tested by applying our techniques to in vivo images of zebrafish as well.

### Illustrating Our Techniques on Wild-Type Zebrafish.

We focus on stripes first because they provide a means of testing our methodology, as wild-type patterns have the most experimental data (collected both in silico and in vivo) available for comparison. Here we use our methodology to evaluate 1,000 wild-type zebrafish patterns generated with the model (20) under the default parameter regime. Previously, model performance (20) was judged by manually counting the number of stochastic simulations that display breaks (or interruptions) in interstripes and requiring matches in pattern features (e.g., number of interstripes present) at major developmental timepoints. In particular, by inspecting 100 in silico patterns, Volkening and Sandstede (20) reported a success rate of 89% according to the former goal, meaning that 89 of 100 simulations had no interruptions in interstripes. (Note that breaks in black stripes are occasionally seen on real fish, so these interruptions were not quantified in ref. 20.) Our methodology allows us to analyze much larger datasets and remove any human error from the process; we demonstrate how topological methods can be used to detect stripe breaks automatically in Fig. 4*A*. Across 1,000 wild-type simulations, we find that 87.5% have no breaks in interstripes (flagged by a decrease in $\beta_{1}$ for $X^{\text{d}}$ cells). This agrees well with the success rate in ref. 20 that was computed using visual inspection.

As an additional evaluation, we manually viewed 200 model outputs and found that the betti numbers capture interstripe breaks with 100% accuracy and only one false positive. In a similar vein, the model (20) was parameterized so that interstripes X1D and X1V (Fig. 1*F*) form between 39 and 44 dpf, but until now this property was judged by visual inspection. Using our automated methods, we show the distribution of times at which these interstripes develop in Fig. 4*B* and find good agreement with the target pattern milestones in ref. 20.

Fig. 4 *C* and *D* shows the distributions of interstripe width and stripe curviness across 1,000 wild-type simulations. The maximum interstripe width, measured by the persistence of the significant dimension 1 persistence points of $X^{\text{l}}$, represents the maximum separation between adjacent stripes. We find that this quantity has a mean of about 814 μm and a SD of approximately 49 μm, which is similar to the average distance between cells (39, 41), suggesting that the average number of cells across the width of a stripe varies by ±1 cell along a stripe. Similarly, in Fig. 4*D*, we show measurements of wild-type stripe curviness (Eq. **2**), a dimensionless quantity that could be compared to empirical data in the future. More generally, Fig. 4 *B* and *D* provides a baseline measurement of the model output (20) that we use to compare to further studies.

### Quantifying “Characteristic” in Noisy Mutant Patterns.

**Fig. 5.**
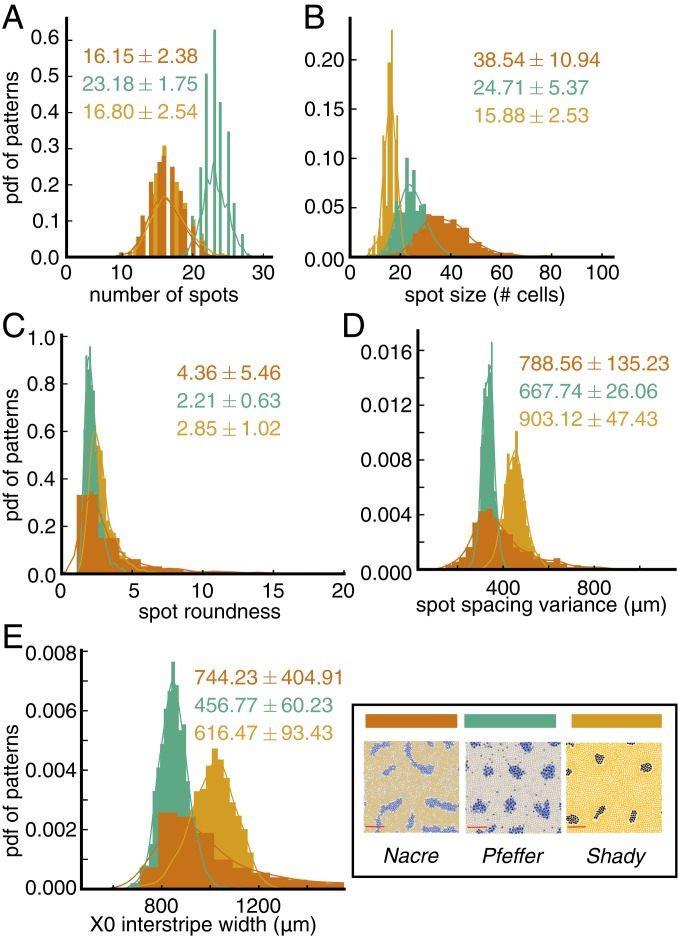
Baseline study of mutant patterns to extract quantifiable features. All measurements are based on 1,000 simulations of the model (20) (for each mutant) under the default parameters. Histograms show distributions for (*A*) the number of spots, (*B*) spot size, (*C*) spot roundness, (*D*) variance in spot spacing, and (*E*) X0 interstripe width (Fig. 1*G*). We overlay KDE curves with a Gaussian kernel on the histograms; the mean plus/minus the SD is shown in each plot for the data.

The *nacre*, *pfeffer*, and *shady* mutants lack specific cell types, leading to altered patterns, which are highly variable and can be broadly described as spotty (Fig. 1 *B*–*D*). Here we use our methods to analyze 1,000 in silico patterns generated with the model (20) under the default parameter regime for each mutant. Our results, shown in Fig. 5, serve as quantitative descriptors of what constitutes “characteristic” for each mutant (according to the model) and demonstrate our methods’ abilities to extract quantifiable differences between spot patterns. Among the three mutants, we find that *pfeffer* has the most spots and that these spots are the most round and the most evenly spaced (Fig. 5 *A* and *C*–*D*). In comparison, *nacre* and *shady* have a similar number of spots, but the spots on *shady* are smaller and rounder than those of *nacre*. (As we noted in *Background and Methods*, we remove a small region at the top and bottom of the domain prior to our analysis to avoid quantifying partial spots.) Moreover, the width of the central X0 interstripe in *pfeffer* is closest to wild-type interstripe width (Fig. 4*C*), while both *nacre* and *shady* feature expanded central interstripes, echoing empirical observations (11). Interestingly, we find that the variance in the number of spots for all three mutants is small (a SD of about two spots). With the exception of *nacre*, which displays the greatest variability in four of the five measurements we present in Fig. 5, the variance in spot spacing and the width of the central interstripe X0 is also small [on the order of the distance between neighboring cells (39)]. In the future, it would be interesting to compare these quantities to large-scale in vivo data and determine what cell interactions in the model (20) are responsible for selecting them robustly.

### Measuring Pattern Variability.

Some cellular interactions on the zebrafish skin are thought to be regulated by direct contact, dendrites, or longer projections (26, 35, 36) (Fig. 1*E*). To account for this, the model (20) assigns disk (short-range communication) and annulus (long-range communication) interaction neighborhoods to each cellular agent (Fig. 1*J*). Cell birth, death, and form transitions are then governed by rules (e.g., Eq. **1**) that depend on the proportion of cells within these neighborhoods. The size of the neighborhoods dictates which cells are able to interact and therefore plays a critical role in patterning. While the interaction neighborhoods have deterministic sizes [based on empirical measurements (26, 36, 39)] in ref. 20, a more realistic model should account for stochastic variations in cell size and projection length. Randomly varying the length scales involved in the interaction neighborhoods serves as a means of including more realistic cellular communication [which could also include diffusion of signaling factors (34) in the future] in agent-based models of zebrafish. As a first step toward including more realistic stochasticity, we therefore replace the deterministic length scales in the model (20) with stochastic length-scale parameters and measure their effect on pattern variability. This models the presence of randomness in cell interactions due to variations in cell size and projection length.

Interaction neighborhoods appear in 17 places in the rules that govern M birth, M death, iridophore form changes, and xanthophore form changes in the model (20). For each cell interaction, we randomly select the size of the associated interaction neighborhood per cell per day from a normal distribution with the mean set to the default parameter value. We vary the SD from 1 to 50% of the mean and for each SD (we consider σ∈{0.01,0.05,0.1,0.2,0.3,0.5}, where σ times the default length scale is the SD of the normal distribution), we run 1,000 simulations each for wild type, *nacre*, *pfeffer*, and *shady*.^#^ Our goal in this study is twofold: First, we aim to make quantitative predictions comparing variability in wild-type and mutant patterns, and second, we seek to identify the range of patterns these fish may display in the presence of stochastic cellular communication.

To quantitatively explore how additional stochasticity impacts patterning, we first need to define what it means for a pattern to look the same as (or different from) what we would expect characteristically. For wild type, this is immediate: We characterize wild-type patterns in terms of stripe and interstripe breaks. For *nacre*, *pfeffer*, and *shady*, however, the process is more challenging because these mutant patterns are messier. For example, from looking at the images of *nacre* in Fig. 1 *B* and *G*, it is not clear at what point in silico patterns consisting of elongated, orange globs should be considered good or bad matches for *nacre*. This is where our baseline analysis of *nacre*, *pfeffer*, and *shady* plays a role. We use our earlier analysis of simulations in the default parameter regime to identify patterns that fall outside of what constitutes “characteristic” for each of these mutants (in terms of number and size of spots). For each mutant, we set our thresholds for small and large spots to be the minimum and maximum values, respectively, of the cluster-size measures that we found in our baseline experiments with that mutant. Analogously, for each mutant, we set the threshold for what constitutes few (many) spots to be the minimum (maximum) number of spots we found in our baseline simulations with that mutant.

**Fig. 6.**
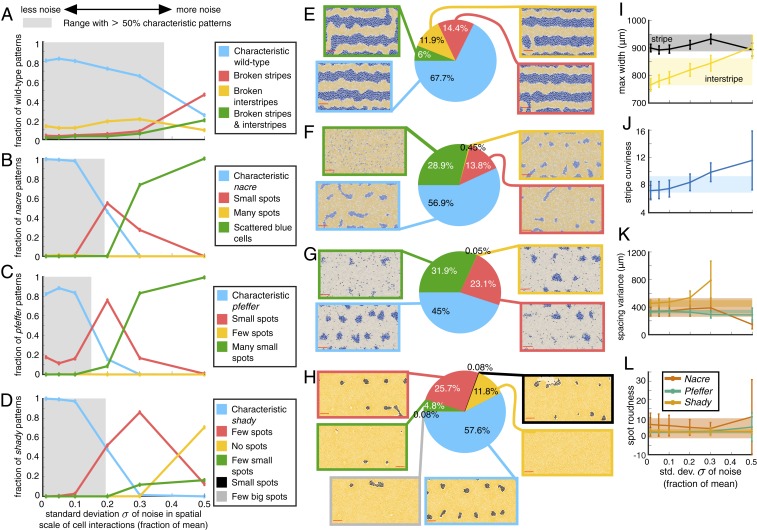
Quantitative study of how stochasticity in cell interactions affects wild-type and mutant zebrafish patterns. For each value of σ∈{0.01,0.05,0.1,0.2,0.3,0.5}, where σ times the default length scale is the SD of the noise that we include in the size of cellular interaction neighborhoods, we analyze 1,000 simulations for wild type and each mutant. (*A–H*) Summary of the patterns that emerge under stochasticity, as detected using our methods for (*A* and *E*) wild type, (*B* and *F*) *nacre*, (*C* and *G*) *pfeffer*, and (*D* and *H*) *shady*. In *A–D*, we highlight the range of σ values that retain at least 50% characteristic patterns under noise in gray. (We define “characteristic” for wild type as patterns having three unbroken interstripes and two unbroken stripes, and we define characteristic for mutants as patterns with spot size and spot number that fall within the baseline distributions in Fig. 5 *A* and *B*.) (*I* and *J*) Mean maximum stripe/interstripe width (*I*) and mean stripe curviness (*J*) for wild type for different noise strengths. (*K* and *L*) Spot spacing variance (*K*) and spot roundness (*L*) for mutants under different noise strengths. In *I–L*, the bars indicate SD and the shaded regions give the characteristic values (the mean ±1 SD) for the associated measurements from our default studies. Also see *SI Appendix*, Tables S2–S5.

In Fig. 6 *A*–*D*, we show how prevalent various patterns are across our stochastic simulations for different levels of noise in cell-interaction length scales (see *SI Appendix*, Tables S2–S5 for additional measurements). As an agglomerate summary across all 6,000 simulations that we generated for different σ values, Fig. 6 *E*–*H* provides examples of the different patterns categorized by our methods for wild type and each mutant. Our results in Fig. 6 *A*–*D* suggest that wild-type and mutant patterns behave differently in the presence of noise. In particular, all three mutants have characteristic spots in less than 50% of the model outputs when σ≥0.2, while wild-type patterns retain characteristic unbroken stripes and interstripes more robustly. If we take a closer look at individual pattern features in Fig. 6 *I* and *J*, we note that low levels of noise (σ≤0.1) serve to straighten stripes and that stripe width is mostly unaffected by the inclusion of noise in cell size and projection length. As stochasticity increases, wild-type patterns display a gradual decay in quality over the range of σ values that we consider. With increasing noise, we find more breaks in interstripes, wider interstripes, curvier stripes, and marginally slower pattern formation (*SI Appendix*, Table S2). Wild-type stripes do not appear to completely deviate from characteristic until σ=0.5, at which point broken stripes become the norm.

In comparison, the mutant patterns are almost unaffected by noise for σ≤0.1, but then undergo a sharp change in pattern features as σ increases. When *nacre* and *pfeffer* stray from characteristic, we mostly observe small spots or scattered cells (Fig. 6 *B* and *C*). Noisy length scales in *shady*, in turn, generally produce patterns with few or no dark spots (Fig. 6*D*). Related, Frohnhöfer et al. (11) observed that strong forms of the *shady* mutant have no spots. As we show in Fig. 6 *B*–*D* and *SI Appendix*, Tables S3–S5, spots on all three mutants retain their characteristic roundness across a range of σ values, deviating substantially from the measures in Fig. 5 only when σ=0.5.

To roughly approximate the amount of noise present in cellular length scales in vivo, we estimate the SD reported for the distance between neighboring xanthophores (33) and the length of their filopodia extensions (30). Based on graphs in ref. 33, we estimate that the distance between the centers of neighboring $X^{\text{d}}$ cells (at 40 dpf) is 27 μm with a SD of 4.6 μm; in our notation, this means that σ=4.6/27, so the SD is about 17% of the mean. Similarly, using graphs in the supporting information of ref. 30, we estimate that the longest xanthophore extensions (measured from the cell center) have a SD in length that corresponds to 12% and 20% of the mean filopodia lengths before and after iridophores arrive on the skin, respectively (in particular, we find that the filopodia length before iridophores arrive is approximately 58 μm ± 6.7 μm, and the filopodia length after iridophores arrive is approximately 25 μm ± 5 μm). These measurements suggest that focusing on the patterns that emerge when σ is between roughly 0.1 and 0.2 in our simulations may have particular biological relevance. We caution that this approximation is based on variance in short-range length scales only, and cells may also communicate through long-range projections (26, 36) [as well as diffusion of signaling molecules (34)]; moreover, in comparing these measurements to our simulations, we are inherently assuming that the empirical data have a normal distribution.

Motivated by our estimates of SD in vivo, we explore what our analysis predicts when σ∈[0.1,0.2]. As we note in *SI Appendix*, Table S2, we find that wild-type stripe width, stripe curviness, and the time of formation of interstripes X1V and X1D are robust in this range of σ. Our methods allow us to estimate that 84.8% and 78.1% of the wild-type patterns for σ=0.1 and σ=0.2, respectively, feature characteristic unbroken interstripes (recall that 87.5% of our simulations in the baseline experiments with σ=0 have unbroken interstripes). Echoing empirical observations (11) that mutant patterns are more variable than wild type, we find that the model (20) supports a distribution of mutant patterns for σ∈[0.1,0.2]. In particular, we predict that the representative images of *nacre*, *pfeffer*, and *shady* in Fig. 1 *B*–*D* and *F*–*H* are characteristic of these mutants in the sense that roughly half of the associated fish may resemble them, while we expect that the remaining fish resemble versions of these images with fewer and smaller spots. Crucially, we predict that the mutants do not commonly display larger spots than those in Fig. 1 *F*–*H*. In the future, analyzing extensive collections of empirical images will allow one to test our predictions and the model (20).

### A Means of Linking Altered Cell Behavior to Mutant Patterns.

Thus far, we have focused on exploring wild-type patterns and the *nacre*, *pfeffer*, and *shady* mutants. Based on transplantation experiments (9–11), these mutant patterns seem to arise because a cell type is missing, rather than due to altered cell interactions. Zebrafish also feature a second type of mutant pattern that forms because cell behavior is altered (often in unknown ways) despite all cell types being present. Examples of this second type of mutant include *leopard* and *obelix*, which feature spots and widened stripes, respectively (9). Mutations that alter cell behavior provide modelers with an opportunity to help link genes to cellular function. [We note that many zebrafish genes have an orthologue in the human genome (50).] One can adjust cell behavior in a model to search for patterns that match various mutants; in this way, a modeling approach can help establish links between cell behaviors and the genes that control them through the phenotype. Agent-based models (e.g., refs. 19–21) often have a large number of parameters, however, and this makes it challenging to comprehensively screen for the cellular interactions that may be related to various mutants by adjusting parameters and visually inspecting the resulting simulations. In a similar vein, modelers seek to present a broad picture of the impact of varying different parameters, but this process is again often limited by the time-consuming nature of visual inspection. We expect that our methods can be used to help address these challenges, and we provide one example to illustrate this process next.

As an example study, we vary a single parameter in the model (20) across a range of values and apply our methods to the resulting patterns. In particular, we focus on the cellular interaction radius represented by $\Omega_{\text{long}}$ in Eq. **1**. As shown in Fig. 1*J* and discussed in *Background and Methods*, long-range interactions depend on the proportion of cells in an annulus region $\Omega_{\text{long}}$ in the model (20). Eq. **1** describes M birth as occurring at randomly selected locations z when the number of $I^{\text{d}}$ and $X^{\text{d}}$ cells in $\Omega_{\text{long}}^{z}$ is sufficiently larger than the number of M in this annulus. This models empirical observations that M differentiate from precursors or stem cells (38, 51, 52) and that $X^{\text{d}}$ and $I^{\text{d}}$ in neighboring interstripes support black cell birth, while other M inhibit it (15, 32) at long range. In ref. 20, the inner radius of the annulus $\Omega_{\text{long}}$ is 210 μm (motivated by in vivo measurements of cellular extensions in refs. 26 and 36) and the width of the annulus is 40 μm. Here we vary the inner radius parameter from 10 to 400 μm in increments of 25 μm and run 100 simulations under each parameter regime for wild type, *pfeffer*, and *shady*^∥^. This allows us to comprehensively explore the impact of long-range signaling on M differentiation.

**Fig. 7.**
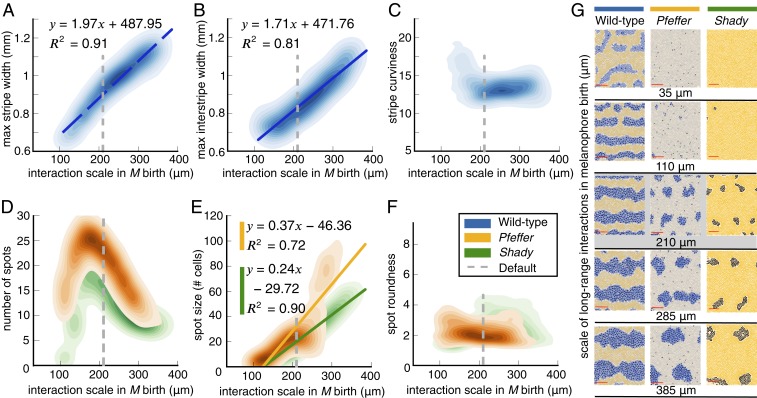
Quantifying in silico pattern dependence on the spatial scale of long-range cellular interactions involved in M birth. (*A–F*) Kernel density estimates for (*A* and *B*) maximum stripe and interstripe width for wild type, (*C*) wild-type stripe curviness, (*D*) number of spots for *pfeffer* and *shady*, (*E*) median spot size for the mutants, and (*F*) *pfeffer* and *shady* spot roundness as a function of the inner radius of the $\Omega_{\text{long}}$ neighborhood in Eq. **1**. Measurements in *A–F* are based on 100 simulations of the model (20) (for wild type, *pfeffer*, and *nacre*, respectively) for each inner radius R of $\Omega_{\text{long}}$ in [**1**] considered. (We consider R from 10 to 400 μm in increments of 25 μm.) All other model parameters (including the width of the $\Omega_{\text{long}}$ annulus in Eq. **1** and the long-range annulus scale in all other model rules) remain at their default values. In *A*, *B*, and *E* we show linear regression models for their corresponding values, along with the $R^{2}$ goodness-of-fit scores. (*G*) Example wild-type, *pfeffer*, and *shady* patterns for different parameter values [the patterns generated by the model (20) under the default parameter—210 μm—are noted in gray].

If $\Omega_{\text{long}}$ in Eq. **1** is too small [e.g., when its inner radius is below 30 to 80 μm, the average distance between cells (33, 39)], it is likely that there are no or very few cells in this annulus region, so that the signal from $X^{\text{d}}$ and $I^{\text{d}}$ to promote M cell birth is effectively turned off. Intuitively, this should lead to an M shortage in the resulting patterns. Conversely, we expect that increasing the inner radius of $\Omega_{\text{long}}$ will widen black stripes. To test these hypotheses and determine the role of this parameter in wild type, we use our methodology to measure stripe width, interstripe width, and stripe curviness across a range of $\Omega_{\text{long}}$ values. For *pfeffer* and *shady*, we compute spot size, number, and roundness as a function of $\Omega_{\text{long}}$ in Eq. **1**. We present our results in Fig. 7 using kernel density estimation plots to visualize the 2D probability density function of pattern features and parameter values.

As we show in Fig. 7 *A*, *B*, and *E*, there is a strong positive correlation between the spatial scale of long-range signaling in M birth and stripe width, interstripe width, and spot size. To check that the quantities we detected automatically agree with results by visual inspection, we show a few sample simulations in Fig. 7*G* for different $\Omega_{\text{long}}$ values. As expected, we find that the width of black stripes in wild type increases as the spatial scale of long-range signals promoting M birth increases. Conversely, when the scale of M-birth signals in Eq. **1** is very local, the resulting patterns vaguely resemble the *nacre* mutant, which features no melanophores (9, 11). This highlights the importance of large-scale simulations and automated methods, as they allow comprehensive model explorations and provide a more complete picture of the roles of different parameters.

To further explore our results, we ran a linear regression analysis on the pattern quantities that we present in Fig. 7. For wild type, we find that a linear model in stripe width yields a coefficient of determination $R^{2}=0.912$, meaning that the linear model captures 91.2% of the observed stripe width variance. For *shady*, a linear model in spot size has a corresponding $R^{2}=0.901$, while a linear model in spot size for *pfeffer* has a lower goodness of fit ($R^{2}=0.722$) because the spot size increases more rapidly. Regression models of this type can be used to predict pattern quantities without needing any reference data. In particular, these simple regression models have the potential to allow one to predict pattern features as a function of cellular interaction signals without needing to perform any model simulations.

The results of our case study exploring the impact of a single parameter (related to long-range signals in melanophore differentiation) show promise. In particular, they suggest that our methods can be applied not only for pattern quantification but also for model sensitivity analysis and large-scale parameter screening to detect possible ways that cell interactions may be altered in mutations. Additionally, we refer to *SI Appendix* for a case study illustrating how our methods can be used to compare and differentiate different zebrafish models. We leave a more thorough investigation of zebrafish mutations and the altered cell interactions involved for future work. For example, the *obelix* mutant (9) features widened stripes due to unknown altered cell interactions; by systematically varying parameters in the model (20) and automatically detecting their impact on stripe width, one could identify a set of altered cell behaviors that may be responsible for this phenotype, and these predictions could then be evaluated experimentally.

## Discussion and Conclusions

Our goal was to provide methods for quantifying agent-based patterns across a range of scales. Leveraging topological data analysis and machine learning, we developed a methodology that captures information spanning local features of interacting cells up to macroscopic spots and stripes. Because it describes shape features across a sequence of spatial scales, persistent homology is a critical tool in our methods. We showed that combining this topological tool with clustering methods yields a collection of summary statistics that can be automatically extracted from patterns using agent coordinates. By reducing the role of visual inspection in describing patterns, our interpretable methodology provides a means of analyzing large datasets and studying how stochasticity in agent interactions affects pattern variability. To illustrate the promise of our methods, we applied our methodology to an extensive dataset of in silico zebrafish skin patterns that we generated using the agent-based model (20). Our methods allowed us to make quantitative predictions about the types and amounts of variability that may arise in wild-type and mutant zebrafish patterns due to stochasticity in cellular communication. We used our methods to distinguish and characterize similar mutant patterns, and we showed how to track pattern features across spatial scales to study the role of different cellular interactions in pattern formation.

Many of our results, which provide a broader view of the agent-based model (20), can be experimentally tested in the future. In particular, after extracting cell coordinates from zebrafish images, one could compute summary statistics for the empirical data and compare these measurements to our simulations. Our methods could also be applied to other models of zebrafish patterning, including partial differential equations (e.g., refs. 8 and 15–17), stochastic cellular automaton perspectives (17, 18), and agent-based models (e.g., refs. 19 and 21). In the future, one could use our methods to optimize model parameters or conduct large screens for cell interactions that may be altered in mutations. Although we focused primarily on analyzing zebrafish patterns at a fixed point in development, future work could track pattern features across developmental timelines.

Our approach to quantifying zebrafish patterns begins to address major challenges associated with quantifying agent-based dynamics in an objective and automated way, but there are also limitations to our methods. First, we make underlying assumptions about the patterns that we are studying. As an example, when we use topological methods to quantify spots or stripes, we assume that the input patterns have certain features (e.g., we assume a wild-type input has stripe patterns). It may be useful for future studies to automatically classify each input pattern as spots or stripes prior to applying the appropriate pattern quantification methods. Moreover, we focused primarily on spots and stripes, but methods for characterizing other patterns [e.g., labyrinth patterns on the *choker* mutant (11)] could be developed in the future. Finally, we note that we built our methodology to take data in the form of agent coordinates. Empirical images and simulations from partial differential equation models, however, are continuous functions defined over 2D domains. In the former case, one option would be to extract cell locations from image data, and, in the latter, one could apply our methods to cell densities after discretizing space and applying a density threshold. Fortunately, functional persistent homology could avoid both of these extra steps as it takes function data as its input. In the future, one could apply our approach to continuous-pattern data by replacing the TDA tools that we used with functional persistence throughout our methodology.

Although we focused on analyzing pattern variability in zebrafish, we expect that a similar approach can be used to quantify agent-based dynamics in other biological settings. Methods that provide summary statistics for pattern features across a range of length scales open up many possibilities for quantitatively comparing large datasets of in silico and in vivo pattern data in the future. By working closely with the needs of each application, we expect that our topological perspective can be extended to analyze agent-based dynamics in wound healing, animal flocks, and other forms of collective behavior.

## Materials and Methods

### Data and Code Availability.

Implementation details and code are freely available on GitHub: https://github.com/sandstede-lab/Quantifying_Zebrafish_Patterns. Simulated data are publicly available on Figshare: https://figshare.com/projects/Zebrafish_simulation_data/72689 (53).

## Supplementary Material

Supplementary File

## Footnotes

*If boundary conditions are not periodic, spots and stripes are topologically equivalent.

^†^Alternatively, we could have used I^d^ and I^l^, but these cells appear closer together than xanthophores at stripe–interstripe boundaries.

^‡^Alternatively, we could have computed the number of spots using X^l^ in *nacre*, as these cells appear together with I^l^. However, X^l^ are much less dense than I^l^ in *nacre*, so the former would introduce more noise into our measurements.

^§^Note that $d_{l_{\infty }}\left(\left(x_{1},y_{1}\right),\left(x_{2},y_{2}\right)\right)=\max\left(\left|x_{1}-x_{2}|,|y_{1}-y_{2}\right|\right)$.

^¶^Alternatively, we could rely on topological summaries to approximate the initial formation of new stripes, but a direct approach is more computationally efficient in this setting.

^#^When we add noise to the annulus parameters, we choose both the inner radius and the annulus width from a normal distribution.

^∥^Note that we do not simulate *nacre* because this mutant has no M cells.
