# Supplementary material for: Topological data analysis of zebrafish patterns
Source: Proc Natl Acad Sci U S A. 2020 Feb 25;117(10):5113–24. doi: 10.1073/pnas.1917763117 (PMC7071871; doi:10.1073/pnas.1917763117)
Supplement: Supplementary File [file pnas.1917763117.sapp.pdf]

PNAS
www.pnas.org

Supplementary Information for

Topological data analysis of zebrafish patterns

Melissa R. McGuirl, Alexandria Volkening, Björn Sandstede

Melissa R. McGuirl.
E-mail: melissa_mcguirl@brown.edu

This PDF file includes:

Supplementary text
Figs. S1 to S8
Tables S1 to S6
References for SI reference citations

www.pnas.org/cgi/doi/10.1073/pnas.1917763117

Supporting Information Text

Additional Background: Persistent Homology. Topological data analysis (TDA) is an emerging branch of applied mathematics that aims to extract useful shape descriptors from large, complex data sets (1–5). The utility of TDA has been demonstrated in a range of applications, including neuroscience (e.g., (6–8)), genomics (e.g., (9–11)), and sensor networks (e.g., (12, 13)). Persistent homology is one of the main techniques in TDA, and it involves the construction of simplicial-complex representations of a given data set across a sequence of scales. We provide an introduction to persistent homology here.

The goal of persistent homology is to associate homology groups to data. For the scope of our paper, we can think of homology groups as vector-space representations of a topological object with dimension corresponding to the number of "holes" that object has. (For more details on algebraic topology, see (14).) Specifically, the 0th and 1st dimension homology groups of an object are vector spaces whose dimensions are the number of connected components and loops, respectively, that the object has. For example, a figure-eight object has a single connected component and two loops (see Fig. 2 in the main manuscript and Fig. S2). In other words, the 0th homology group of a figure eight has one generator, and its first homology group has two generators.

Next consider a data set $S = \{x_i\}_{i \in I}$, where S is any collection of points living in a given metric space (D, d). For example, S could be a set containing the coordinates of all of the pigment cells in a zebrafish skin pattern. If we were to extract the homology groups of S directly, we would get uninformative representations. In particular, the 0th homology group of S has dimension equal to the number of data points in S and all of the higher homology groups are trivial (e.g., if S contained the coordinates of pigment cells, the 0th homology group of S would simply be the number of cells in S). Instead, in persistent homology, one builds simplicial-complex representations of S to infer the homology of the manifold from which the data were sampled. In our example, this allows us to study the qualities of the stripes and spots that are made up of the pigment cell coordinates in S.

A k-simplex of $k+1$ affinely independent points is the convex polygon whose vertices are precisely the $k+1$ affinely independent points (3, 4). In other words, a 0-simplex $\sigma_0(x_1)$ is a single point x_1; a 1-simplex $\sigma_1(x_1, x_2)$ is an edge connecting x_1 and x_2; a 2-simplex $\sigma_2(x_1, x_2, x_3)$ is a filled triangle whose vertices are x_1, x_2, and x_3; and so on. To begin building simplicial-complex representations of S, we place a ball of radius r centered at each $x_i \in S$ to obtain $\{B_r(x_i) = \{y \in D : d(x_i, y) \leq r\}\}_{i \in I}$. The simplicial complex representation of S with respect to r is the union of all k-simplices $\sigma_k(x_{j_0}, \ldots, x_{j_k})$ such that $B_r(x_{j_l}) \cap B_r(x_{j_m}) \neq \emptyset$ for all $l, m = 0, 1, \ldots, k$. This is known as the Vietoris–Rips complex of S with respect to r. We can now compute the homology groups of this simplicial-complex representation of S. See Fig. S1 for examples of different simplicies.

Inevitably, the homology groups of the simplicial-complex representation of S will be sensitive to the choice of scaling parameter r. To overcome this challenge, persistent homology instead tracks how the homology groups change across an increasing sequence of scaling parameters $\{r_j\}_{j \in J}$. The scaling parameter at which a homological generator appears is called the birth radius (r_b) of a topological feature. Similarly, the value of r at which a homological generator disappears is called the death radius (r_d) of a topological feature. The persistence of a topological feature is its lifetime, namely $r_d - r_b$.

In TDA there are two primary ways of visualizing the persistent homology of a data set. The first method, called a barcode diagram, is a collection of bars. Each bar in a barcode diagram represents a topological generator; the left endpoint of a bar corresponds to the birth radius of that generator, and the right endpoint of a bar corresponds to its death radius. Topological features that persist for across a longer range of radii values are identified by having longer bar representations in the barcode diagram. The second method, called a persistence diagram, is a collection of points in $\mathbb{R}^2_{\geq 0}$. Each point in a persistence diagram corresponds to a topological feature, and the x- and y-coordinates of the points are the birth and death radii, respectively, of those features. In a barcode diagram, topological features that persist across a broader range of parameter values r lie farthest away from the diagonal line $y = x$. These persistent homology visualizations provide a low-dimensional, descriptive representation of the original data S and are therefore useful for a range of data-driven tasks. For example, in Fig. S2F–G we show the dimension 0 and dimension 1 barcode diagrams corresponding to the figure-eight example in the main text (see Fig. 2 in the main text). We show the analogous persistence diagram for this example in Fig. S2H. The long bar in Fig. S2F represents the single connected component of the figure-eight shape and the two long bars in Fig. S2G correspond to the two loops of the figure-eight shape. In the persistence diagram, these topological features are represented by the top left circular teal point in Fig. S2H (dimension 0 feature) and the two triangular pink points in the top left corner in Fig. S2H (dimension 1 features).

Additional Background: Model Details. To demonstrate our TDA-based methods and study variability in zebrafish patterns, we generate *in silico* data by simulating the agent-based model (15). Here we provide further background on this model, highlighting where it is descriptive or predictive (see (15) for full details and Figure S3 for an overview). The model (15) treats pigment cells as individual agents of five types and tracks their coordinates on growing 2–D domains that represent a third of the fish body (Figure S3A–B). In particular, we let

$$\begin{aligned}
\mathbf{M}_i(t) &= \text{ position of the } i\text{th black melanophore at time } t; \\
\mathbf{X}_i^{\mathrm{d}}(t) &= \text{position of the } i\text{th orange dense xanthophore at time } t; \\
\mathbf{X}_i^{\mathrm{l}}(t) &= \text{position of the } i\text{th yellow loose xanthophore at time } t; \\
\mathbf{I}_i^{\mathrm{d}}(t) &= \text{position of the } i\text{th silver dense xanthophore at time } t; \text{ and} \\
\mathbf{I}_i^{\mathrm{l}}(t) &= \text{position of the } i\text{th blue loose iridophore at time } t.
\end{aligned}$$

There are four types of cellular interactions: movement, differentiation (or birth), competition, and transitions in agent type/form between dense and loose (15). For the former three types of interactions, the model (15) is largely descriptive of what is currently known in the biological literature. Additionally, some of the cellular dynamics involved in instructing xanthophores to change their form became better understood during model development (15, 16). In contrast, the model (15) predicts the unknown cues (at a phenomenological level) that drive iridophores to change their form by requiring consistency with a broad range of mutant patterns and experiments.

Each cellular agent moves according to an ordinary differential equation that includes repulsion from or attraction to other cells (Figure S3D) (15). For example, the M cell at position $\mathbf{M}_i(t)$ moves according to the following equation, which we reproduce from (15):

$$\frac{d\mathbf{M}_i}{dt} = \sum_{j=1, j\neq i}^{N_\mathrm{M}} f^{\mathrm{MM}}(||\mathbf{M}_j - \mathbf{M}_i||)\frac{\mathbf{M}_j - \mathbf{M}_i}{||\mathbf{M}_j - \mathbf{M}_i||} + \sum_{j=1}^{N_\mathrm{X}^\mathrm{d}} f^{\mathrm{X^dM}}(||\mathbf{X}_j^\mathrm{d} - \mathbf{M}_i||)\frac{\mathbf{X}_j^\mathrm{d} - \mathbf{M}_i}{||\mathbf{X}_j^\mathrm{d} - \mathbf{M}_i||} + \sum_{j=1}^{N_\mathrm{I}^\mathrm{d}} f^{\mathrm{I^dM}}(||\mathbf{I}_j^\mathrm{d} - \mathbf{M}_i||)\frac{\mathbf{I}_j^\mathrm{d} - \mathbf{M}_i}{||\mathbf{I}_j^\mathrm{d} - \mathbf{M}_i||},$$

where N_M, N_X^d, and N_I^d denote the numbers of M, X^d, and I^d cells, respectively. The parameters in the functions f^{**}, which allow for local interactions between cells, are fit to measurements of cell–cell distances and cell speed (e.g., (17, 18)), and are motivated by observations of the directions that cells move relative to their neighbors (e.g., (17, 19)).

In the model (15), cell birth, competition, and changes in form depend on the agents in five interaction neighborhoods, which capture short- and long-range dynamics:

$$B_{75}^{\mathbf{z}} = \text{disk of radius 75 } \mu\text{m with its center at position } \mathbf{z}; \quad \text{[S1]}$$

$$B_{\Delta_\mathrm{xm}}^{\mathbf{z}} = \text{disk of radius } \Delta_\mathrm{xm} = 82 \ \mu\text{m with its center at position } \mathbf{z}; \quad \text{[S2]}$$

$$B_{90}^{\mathbf{z}} = \text{disk of radius 90 } \mu\text{m with its center at position } \mathbf{z}; \quad \text{[S3]}$$

$$B_{90/2}^{\mathbf{z}} = \text{disk of radius } 90/2 = 45 \ \mu\text{m with its center at position } \mathbf{z}; \text{ and} \quad \text{[S4]}$$

$$\Omega_\mathrm{long}^{\mathbf{z}} = \text{annulus of inner radius 210 } \mu\text{m and width 40 } \mu\text{m with its center at position } \mathbf{z}, \quad \text{[S5]}$$

where $\mathbf{z}$ is position of the cell (or precursor) of interest (Figure S3C). These length scales are based on measurements of cell–cell distances and cellular extension lengths (16, 17, 20–22). For example, the average distances between melanophores and xanthophores at stripe–interstripe boundaries is 82 μm (17).

The authors (15) specify that new xanthophores and iridophores appear on the domain through division of existing cells when there is space. Melanophore birth, in comparison, occurs at random locations (modeling birth from precursors (23–25)) due to long-range signals from X^d and I^d cells (26, 27). In particular, as we note in Eq. (1) in the main text, a new M cell appears at the randomly-selected (and not already overcrowded) position $\mathbf{z}$ if there is a sufficient number of I^d and X^d cells in $\Omega_\mathrm{long}^{\mathbf{z}}$ (see Eq. (S5)) (15). The model (15) also includes local competition between dense xanthophores and melanophores, as well as long-range survival cues from X^d to M. Importantly, the model's rules and the length scales involved for all of these interactions (Figure S3E) are based on empirical observations (e.g., (25–30)). To determine parameters, the authors fit the model to cell–cell distance measurements (16–18), rough measurements of stripe width, and the major timepoints in pattern formation (e.g., when a new interstripe appears) (15).

Lastly, the model (15) allows xanthophores and iridophores to change their form (e.g., agent type and, thus, the rules governing their behavior) between dense and loose. In agreement with empirical observations (16), xanthophores change their form based on local signals (Figure S3F). For example, I^d cells express a factor (called Csf1) that promotes X^d *in vivo* (26, 31). Because iridophore-form transitions are not yet well-understood empirically, the authors (15) identified a set of possible mechanisms that may govern these dynamics. To determine the unknown signals involved in iridophore-form transitions, they fit the model to wild-type, *pfeffer*, *nacre*, and *shady* pattern development. Providing support for the proposed rules (Figure S3G), the model reproduces a range of experiments (not used for model fitting), as well as the skin patterns on two evolutionary relatives of zebrafish (15).

Summary of Model Rules Adjusted in our Study of Pattern Variability. In Fig. 6 in the main text, we quantify the pattern variability induced by replacing the original deterministic length scales used in the model (15) (see Eq. (S1)–Eq. (S5) above) with stochastic length scales. With the exception of $B_{\Delta_\mathrm{xm}}^{\mathbf{z}}$, which is used in the rules that prevent cell overcrowding due to continual birth, we replace these deterministic neighborhoods with stochastic versions to generate our results in Fig. 6 in the main text, as follows:

$$B_{75,\sigma}^{\mathbf{z}} = \text{disk centered at } \mathbf{z} \text{ with radius } r \sim \mathcal{N}(75, \sigma\cdot 75) \ \mu\text{m}; \quad \text{[S6]}$$

$$B_{90/2,\sigma}^{\mathbf{z}}, B_{90,\sigma}^{\mathbf{z}} = \text{disks centered at } \mathbf{z} \text{ with radii } r/2 \text{ and } r, \text{ respectively, where } r \sim \mathcal{N}(90, \sigma\cdot 90) \ \mu\text{m}; \quad \text{[S7]}$$

$$B_{90/2,\sigma}^{\mathbf{z}} = \text{disk centered at } \mathbf{z} \text{ with radius } r/2,\ r \sim \mathcal{N}(90, \sigma\cdot 90) \ \mu\text{m; and} \quad \text{[S8]}$$

$$\Omega_{\mathrm{long},\sigma}^{\mathbf{z}} = \text{annulus centered at } \mathbf{z} \text{ with inner radius } r \sim \mathcal{N}(210, \sigma\cdot 210) \ \mu\text{m and width } w \sim \mathcal{N}(40, \sigma\cdot 40) \ \mu\text{m}, \quad \text{[S9]}$$

where we consider $\sigma \in \{0.01, 0.05, 0.1, 0.2, 0.3, 0.5\}$, so that the standard deviation of the noise is a fraction of its mean.* We write $B_{90/2,\sigma}^{\mathbf{z}}$ instead of $B_{45,\sigma}^{\mathbf{z}}$ to stress that, when we randomly select $r \sim \mathcal{N}(90, \sigma\cdot 90)$ μm, we use that same r value for both

*Note that $\sigma = 0$ corresponds to the deterministic length scales originally used in the model (15).

the radius of the disk $B^{\mathbf{z}}_{90,\sigma}$ and the diameter of the disk $B^{\mathbf{z}}_{90/2,\sigma}$. This means that the radius of $B^{\mathbf{z}}_{90/2,\sigma}$ is always half the radius of $B^{\mathbf{z}}_{90,\sigma}$ for the cell at position $\mathbf{z}$ (we made this choice because the size of these disks depends on a single parameter in the model (15)). In all other cases, following the approach in (15), the length scales involved in the disk and annulus neighborhoods are chosen independently.

We include these stochastic cell-interaction neighborhoods in the model rules for M birth, M death, and cell-form transitions. Because it makes the model more amenable to testing a wide range of length scales, we also reframe the model rules in (15) so that the rules for M birth, M death, and xanthophore form transitions depend on the ratios of cell counts in the interaction neighborhoods rather than absolute cell counts, and the rules for iridophore form transitions depend on density counts. While ideally all rules would be modified to be in terms of density counts, this is only feasible for the iridophore form transition rules that have only one interaction neighborhood per inequality.

We present the adjusted model rules that we use to generate our results in Fig. 6 in the main text in Eq. (S10)–Eq. (S15) below. We refer to (15) for their biological motivation and summarize all of the model rules pictorially in Fig. S3; for each rule, we select each stochastic length scale (namely, Eq. (S6)–Eq. (S9)) randomly per cell per day (i.e., the time step for cell interactions used in (15)). In all other cases, the parameters have the default values given in (15). Specifically, we adapt the model rule for M birth at position $\mathbf{z}$ from (15) to the following:

$$\underbrace{\frac{\sum_{i=1}^{N_{\mathrm{X}}^{\mathrm{d}}} \mathbb{1}_{\Omega^{\mathbf{z}}_{\mathrm{long},\sigma}}(\mathbf{X}^{\mathrm{d}}_i) + \sum_{i=1}^{N_{\mathrm{I}}^{\mathrm{d}}} \mathbb{1}_{\Omega^{\mathbf{z}}_{\mathrm{long},\sigma}}(\mathbf{I}^{\mathrm{d}}_i)}{\alpha + \beta \sum_{i=1}^{N_{\mathrm{M}}} \mathbb{1}_{\Omega^{\mathbf{z}}_{\mathrm{long},\sigma}}(\mathbf{M}_i)} > 1}_{\text{long-range signals for melanophore birth}} \quad \text{and}$$

$$\underbrace{\sum_{i=1}^{N_{\mathrm{X}}^{\mathrm{d}}} \mathbb{1}_{B^{\mathbf{z}}_{\Delta_{\mathrm{xm}}}}(\mathbf{X}^{\mathrm{d}}_i) + \sum_{i=1}^{N_{\mathrm{M}}} \mathbb{1}_{B^{\mathbf{z}}_{\Delta_{\mathrm{xm}}}}(\mathbf{M}_i) + \sum_{i=1}^{N_{\mathrm{I}}^{\mathrm{d}}} \mathbb{1}_{B^{\mathbf{z}}_{\Delta_{\mathrm{xm}}}}(\mathbf{I}^{\mathrm{d}}_i) \leq \eta}_{\text{limiting condition to prevent overcrowding}} \quad \Longrightarrow \quad \text{melanophore birth at } \mathbf{z}, \qquad \text{[S10]}$$

where $\mathbb{1}_R(\mathbf{x})$ with $R \in \{B^{\mathbf{z}}_{90/2,\sigma}, B^{\mathbf{z}}_{75,\sigma}, B^{\mathbf{z}}_{\Delta_{\mathrm{xm}}}, B^{\mathbf{z}}_{90,\sigma}, \Omega^{\mathbf{z}}_{\mathrm{long}}\}$ is the indicator function for the region R (in particular, $\mathbb{1}_R(\mathbf{x}) = 1$ if $\mathbf{x}$ is in the region R and 0 otherwise). Our adapted rule for melanophore death due to local competition (15, 27), in turn, is given by:

$$\frac{\sum_{j=1}^{N_{\mathrm{X}}^{\mathrm{d}}} \mathbb{1}_{B^{\mathbf{M}_j}_{90,\sigma}}(\mathbf{X}^{\mathrm{d}}_j)}{\sum_{j=1}^{N_{\mathrm{M}}} \mathbb{1}_{B^{\mathbf{M}_i}_{90,\sigma}}(\mathbf{M}_i)} > \mu \implies \text{death of melanophore at } \mathbf{M}_i \text{ due to local competition with } X^{\mathrm{d}}. \qquad \text{[S11]}$$

The model (15) also includes a rule that M cells may die due to the absence of long-range signals that are necessary for their survival (27) when blue I^{l} cells are not present nearby. We adapt this rule as follows:

$$\frac{\sum_{j=1}^{N_{\mathrm{M}}} \mathbb{1}_{\Omega^{\mathbf{M}_i}_{\mathrm{long},\sigma}}(\mathbf{M}_i)}{\sum_{i=1}^{N_{\mathrm{X}}^{\mathrm{d}}} \mathbb{1}_{\Omega^{\mathbf{M}_i}_{\mathrm{long},\sigma}}(\mathbf{X}^{\mathrm{d}}_j)} \geq \xi \quad \text{and} \quad \sum_{j=1}^{N_{\mathrm{I}}^{\mathrm{d}}} \mathbb{1}_{B^{\mathbf{M}_i}_{90/2,\sigma}}(\mathbf{I}^{\mathrm{l}}_j) < \nu \implies \text{death of cell at } \mathbf{M}_j \text{ with probability } p_{\mathrm{death}} \text{ per day.}$$

In the same way, we adapt the model rule for xanthophore-form transitions (see Fig. S3F) from (15) by replacing the deterministic interaction neighborhoods in the original rules with their stochastic equivalents and reframing the rules in terms of ratios:

$$\frac{\sum_{j=1}^{N_{\mathrm{I}}^{\mathrm{l}}} \mathbb{1}_{B^{\mathbf{X}^{\mathrm{d}}_i}_{75,\sigma}}(\mathbf{I}^{\mathrm{l}}_j)}{a + \sum_{j=1}^{N_{\mathrm{I}}^{\mathrm{d}}} \mathbb{1}_{B^{\mathbf{X}^{\mathrm{d}}_i}_{90/2,\sigma}}(\mathbf{I}^{\mathrm{d}}_j)} > 1 \implies \mathbf{X}^{\mathrm{d}}_i \text{ becomes loose,} \qquad \text{[S12]}$$

$$\frac{\sum_{j=1}^{N_{\mathrm{I}}^{\mathrm{d}}} \mathbb{1}_{B^{\mathbf{X}^{\mathrm{l}}_i}_{90/2,\sigma}}(\mathbf{I}^{\mathrm{d}}_j) + P_i \sum_{j=1}^{N_{\mathrm{X}}^{\mathrm{d}}} \mathbb{1}_{B^{\mathbf{X}^{\mathrm{l}}_i}_{75,\sigma}}(\mathbf{X}^{\mathrm{d}}_j)}{b + \sum_{j=1}^{N_{\mathrm{I}}^{\mathrm{l}}} \mathbb{1}_{B^{\mathbf{X}^{\mathrm{l}}_i}_{90/2,\sigma}}(\mathbf{I}^{\mathrm{l}}_j) + \sum_{j=1}^{N_{\mathrm{M}}} \mathbb{1}_{B^{\mathbf{X}^{\mathrm{l}}_i}_{90,\sigma}}(\mathbf{M}_j)} > 1 \implies \mathbf{X}^{\mathrm{l}}_i \text{ becomes dense.} \qquad \text{[S13]}$$

Lastly, we adapt the rules proposed in (15) for iridophore form transitions between dense and loose (Fig. S3G) by rescaling

by the area of the interaction neighborhoods so that these rules are now in terms of density counts as follows:

$$\left(\frac{1}{\left|B^{\mathbf{I}_i^{\mathrm{l}}}_{90,\sigma}\right|}\sum_{j=1}^{N_{\mathrm{M}}}\mathbb{1}_{B^{\mathbf{I}_i^{\mathrm{l}}}_{90,\sigma}}(\mathbf{M}_j) < \frac{c}{\left|B^{\mathbf{I}_i^{\mathrm{l}}}_{90}\right|} \text{ and } \frac{1}{\left|\Omega^{\mathbf{I}_i^{\mathrm{l}}}_{\mathrm{long},\sigma}\right|}\sum_{j=1}^{N_{\mathrm{X}}^{\mathrm{d}}}\mathbb{1}_{\Omega^{\mathbf{I}_i^{\mathrm{l}}}_{\mathrm{long},\sigma}}(\mathbf{X}_j^{\mathrm{d}}) < \frac{d}{\left|\Omega^{\mathbf{I}_i^{\mathrm{l}}}_{\mathrm{long}}\right|}\right) \text{ or}$$

$$\left(\frac{1}{\left|B^{\mathbf{I}_i^{\mathrm{l}}}_{90,\sigma}\right|}\sum_{j=1}^{N_{\mathrm{M}}}\mathbb{1}_{B^{\mathbf{I}_i^{\mathrm{l}}}_{90,\sigma}}(\mathbf{M}_j) < \frac{c}{\left|B^{\mathbf{I}_i^{\mathrm{l}}}_{90}\right|} \text{ and } \frac{1}{\left|B^{\mathbf{I}_i^{\mathrm{l}}}_{75,\sigma}\right|}\sum_{j=1}^{N_{\mathrm{X}}^{\mathrm{d}}}\mathbb{1}_{B^{\mathbf{I}_i^{\mathrm{l}}}_{75,\sigma}}(\mathbf{X}_j^{\mathrm{d}}) > \frac{e}{\left|B^{\mathbf{I}_i^{\mathrm{l}}}_{75}\right|}\right) \implies \mathbf{I}_i^{\mathrm{l}} \text{ transforms to dense,} \quad \text{[S14]}$$

$$\left(\frac{1}{\left|B^{\mathbf{I}_i^{\mathrm{d}}}_{90,\sigma}\right|}\sum_{j=1}^{N_{\mathrm{M}}}\mathbb{1}_{B^{\mathbf{I}_i^{\mathrm{d}}}_{90,\sigma}}(\mathbf{M}_j) > \frac{f}{\left|B^{\mathbf{I}_i^{\mathrm{d}}}_{90}\right|}\right) \text{ or}$$

$$\left(\frac{1}{\left|\Omega^{\mathbf{I}_i^{\mathrm{d}}}_{\mathrm{long},\sigma}\right|}\sum_{j=1}^{N_{\mathrm{X}}^{\mathrm{d}}}\mathbb{1}_{\Omega^{\mathbf{I}_i^{\mathrm{d}}}_{\mathrm{long},\sigma}}(\mathbf{X}_j^{\mathrm{d}}) > \frac{g}{\left|\Omega^{\mathbf{I}_i^{\mathrm{d}}}_{\mathrm{long}}\right|} \text{ and } \frac{1}{\left|B^{\mathbf{I}_i^{\mathrm{d}}}_{75,\sigma}\right|}\sum_{j=1}^{N_{\mathrm{X}}^{\mathrm{d}}}\mathbb{1}_{B^{\mathbf{I}_i^{\mathrm{d}}}_{75,\sigma}}(\mathbf{X}_j^{\mathrm{d}}) < \frac{h}{\left|B^{\mathbf{I}_i^{\mathrm{d}}}_{75}\right|}\right) \implies \mathbf{I}_i^{\mathrm{d}} \text{ transforms to loose,} \quad \text{[S15]}$$

where $|R|$ denotes the area of the interaction neighborhood R.

Measuring Local Pattern Features. While TDA and machine learning offer exciting insights into biological patterns, raw calculations of agent–agent distances and agent-density counts are still important measurements. We therefore use direct calculations in tandem with the aforementioned methods for tracking pattern variability. In particular, we calculate the mean and variance of nearest-neighbor distances between agents directly using location data. The coefficient of variation (CV) for the distance between neighboring agents provides an additional measurement of pattern quality:

$$\mathrm{CV} = 100 \times \frac{\mathrm{std(agent–agent\ distances)}}{\mathrm{mean(agent–agent\ distances)}}. \quad \text{[S16]}$$

For zebrafish, low values of the M CV are associated with better-formed patterns (18, 32). We compute A–B agent density counts by counting the number of agents of type B per local neighborhood of the query agent type A, where local neighborhoods are defined as disks of radius R centered at the query agent (we use $R = 250$ μm for X^{l} cells and $R = 200$ μm for I^{l} cells). We then use the 20th percentile of agent counts across all local neighborhoods as our summary statistic (other summary statistics could also be used). It is helpful to note that the A–A agent density is just the traditional measurement of the density of agent A, but we find that extending this definition to allow for counting one agent type against another is helpful in some cases. For example, in the *pfeffer* mutant pattern (see Fig. 1C and 1G in the main manuscript), blue I^{l} cells are present only when there are sufficient black M nearby. To calculate the I^{l}–M density, we count the number of M cells within a local neighborhood of each I^{l} cell and take a summary measure across all neighborhoods.

We present the M CV and nearest-neighbor cell–cell spacing measurements from 1,000 model simulations under the default parameter regime for wild-type, *nacre*, *pfeffer*, and *shady* in Fig. S4, and we show X^{l}–M and I^{l}–M cell-density counts in Fig. S5. We observe that the *shady* mutant has the largest variability in M CV (Fig. S4A). Moreover, the average M–M nearest-neighbor spacing is smallest for *shady* and greatest for *pfeffer* (Fig. S4B). Interestingly, the distributions of the mean X^{d}–X^{d} nearest-neighbor spacing are completely disjoint for wild-type, *nacre*, and *shady*. The wild-type X^{d}–X^{d} nearest-neighbor spacing is the smallest and the *shady* X^{d}–X^{d} nearest-neighbor spacing is the largest, with all three types having low variance (Fig. S4C). In contrast, the wild-type M–X^{d} nearest-neighbor spacing is significantly greater than the *shady* M–X^{d} nearest-neighbor spacing (Fig. S4E). Finally, we note that the X^{l}–M (Fig. S5A) and I^{l}–M (Fig. S5B) density counts are greater for wild-type than they are for the mutants.

Example Application: Distinguishing between Different Models. To further demonstrate the utility of TDA-based methods for quantifying zebrafish patterns, we apply the methods presented in this paper to compare multiple models for skin pattern formation. Here we study two alternative models that we obtained by changing parameters in the agent-based model (15) to produce different types of stripe patterns.

First, as a preliminary study of pattern variability we changed the six length scale parameters involved in iridophore-form transitions. We focus on iridophore-form transitions because these dynamics are not well-understood biologically, as we discussed above. In particular, we considered $B_{90}^{\mathbf{I}_i^{\mathrm{d}}}, B_{90}^{\mathbf{I}_i^{\mathrm{l}}} \in [10, 170]$, $B_{75}^{\mathbf{I}_i^{\mathrm{d}}}, B_{75}^{\mathbf{I}_i^{\mathrm{l}}} \in [10, 140]$, and $\Omega_{\mathrm{long}}^{\mathbf{I}_i^{\mathrm{d}}}, \Omega_{\mathrm{long}}^{\mathbf{I}_i^{\mathrm{l}}} \in [10, 410]$, and then selected 200 points randomly in this six-dimensional parameter space. After running each of these models, we selected a model that maintained stripe patterns but whose simulations appeared to feature thinner interstripes with more interstripe breaks, in comparison to model simulations from (15) for wild-type zebrafish. Specifically, the first new model we chose for this comparative study, which we will refer to as alternative model I, has the following parameter values: $B_{90}^{\mathbf{I}_i^{\mathrm{d}}} = 115$ μm, $B_{75}^{\mathbf{I}_i^{\mathrm{d}}} = 25$ μm, $\Omega_{\mathrm{long}}^{\mathbf{I}_i^{\mathrm{d}}} = 230$ μm, $B_{90}^{\mathbf{I}_i^{\mathrm{l}}} = 130$ μm, $B_{75}^{\mathbf{I}_i^{\mathrm{l}}} = 10$ μm, and $\Omega_{\mathrm{long}}^{\mathbf{I}_i^{\mathrm{l}}} = 190$ μm (with all other parameters set to the default values in (15)). As with the original model (15), alternative model I has deterministic length-scale parameters so that the length scales involved in cell interactions are constant for each cell across time. Notably, alternative model I includes the same cellular

mechanisms, governing equations, and stripe-like outputs as the original model (15), and the two models differ only in the magnitude of the length scales that appear in the rules for iridophore-form transitions (Fig. S3G). By comparing the patterns generated by these two models with largely overlapping properties, we will be able to test the sensitivity of our methods in distinguishing between models that produce qualitatively similar patterns.

Second, we modified the original model (15) to determine the effects of removing a single cellular interaction from the model. Namely, in alternative model II, we remove the weak attraction of X^{d} to I^{d} cells (see Fig. S3D). We compare this alternative model to the original model (15) to test if removing a single mechanism causes detectable and quantifiable pattern differences.

For our analysis, we generate $1,000$ simulations of each alternative model, and we compare these results to $1,000$ simulations of wild-type zebrafish patterns generated with the model (15) under its default parameter regime. Patterns simulated under alternative model I feature thin, curvy and broken yellow interstripes with wider dark stripes, whereas patterns simulated under alternative model II have wide yellow interstripes with thinner dark stripes, resembling the *idefix* zebrafish mutant (33). We show example model outputs from alternative model I and alternative model II in Fig. S6.

In quantifying the patterns from alternative model I, we identified that 96.1% of the results had yellow interstripe breaks, 3.7% of the model simulations featured no stripe or interstripe breaks, and 0.2% of the model simulations had breaks in both stripes and interstripes (see Fig. S7A). For alternative model II, in contrast, 42.5% of the model simulations had no stripe or interstripe breaks, while 46.2% of the simulations had stripe breaks. In addition, 2.7% of the alternative model II simulations had interstripe breaks, and 8.6% of the simulations featured breaks in both stripes and interstripes (see Fig. S7B). In comparison, as shown in Fig. 4A in the main manuscript, across our simulations of the original model (15), 83.6% of its wild-type patterns had no stripe or interstripe breaks, 10.7% contained interstripe breaks, 3.9% had stripe breaks, and 1.8% contained breaks in both stripes and interstripes. This suggests that the topological features used for characterizing stripe properties can differentiate between stripes generated from models with varying length scales and altered cellular interactions.

For the original model and two alternatives, we show distributions of their pattern features that we quantified using our methods in Fig. S8. As we show in Fig. S8A–B, our methods for quantifying stripe and interstripe width differentiate between the patterns generated under these three different models. In contrast, our methods for quantifying stripe curviness appear to distinguish alternative model I from both the original model and alternative model II, but there is strong overlap between the corresponding distributions from the original model and alternative model II (see Fig. S8C). We note, however, that some of the values for our stripe curviness measure for alternative model I in Fig. S8C are negative. The patterns simulated from alternative model I contain a high percentage of yellow interstripe breaks and occasionally resemble spot-like features aligned in stripes. These stripe breaks make accurately detecting stripe boundaries increasingly difficult, resulting in erroneous stripe curviness calculations. It will be useful for future research to focus on improving our ability to accurately calculate stripe curviness in the presence of highly-broken stripes.

Next, as we show in Fig. S8D, there is significant overlap between the distributions of mean M–M spacing from the original model and alternative model I, but the mean M–M spacing for alternative model II is lower. Moreover, the distributions of mean X^{d}–X^{d} spacing are completely disjoint for all three models (Figure S8E), while the corresponding distributions of mean X^{l}–X^{l} spacing overlap slightly (Fig. S8F).

Lastly, we ran two-sided Kolmogorov–Smirnov tests (in Python 3.0 using Scipy) on the distributions of pattern features for each of the three models under consideration. With two exceptions (namely, comparing stripe curviness in the original model and alternative model II, and comparing mean M–M spacing in the original model and alternative model I), the Kolmogorov–Smirnov test results indicate that the differences between the distributions of stripe features for each of the three models in Fig. S8 are statistically significant. See Table S6 for the exact test statistics and p-values. These results demonstrate that our methods can automatically distinguish between models that produce different types of stripe patterns by comparing quantifiable features and testing for statistical significance. Future work can build upon these results by comparing multiple models to experimental data in an effort to select the most accurate model for simulating zebrafish pattern formation.

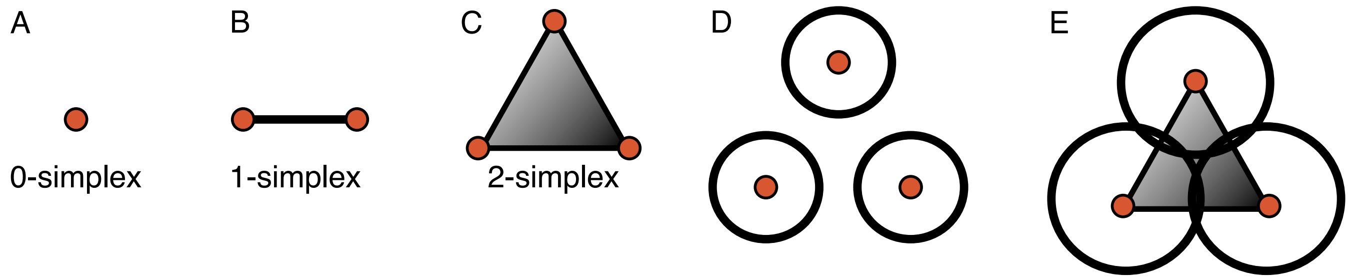

Fig. S1. Introduction to simplicial complexes. Examples of (A) a 0-simplex (point), (B) a 1-simplex (edge or line), and (C) a 2-simplex (triangle). (D–E) Given 3 points (namely, x_1, x_2, and x_3), we show their Vietoris–Rips simplicial complex with respect to two separate radius parameters. In panel (D), the radius parameter r is small enough so that $B_r(x_i) \cap B_r(x_j) = \emptyset$ for all $i, j = 1, 2, 3$, $i \neq j$. Consequently, the Vietoris–Rips complex with respect to the r value in (D) simply consists of three 0-simplices: $\sigma_0(x_1)$, $\sigma_0(x_2)$, and $\sigma_0(x_3)$. As the radius parameter r grows in panel (E), we find pairwise non-empty intersections $B_r(x_1) \cap B_r(x_2) \neq \emptyset$, $B_r(x_1) \cap B_r(x_3) \neq \emptyset$, and $B_r(x_2) \cap B_r(x_3) \neq \emptyset$, so the resulting Vietoris–Rips simplicial complex is the union of three 0-simplices $\sigma_0(x_1)$, $\sigma_0(x_2)$, and $\sigma_0(x_3)$; three 1-simplices $\sigma_1(x_1, x_2)$, $\sigma_1(x_1, x_3)$, and $\sigma_1(x_2, x_3)$; and one 2-simplex $\sigma_2(x_1, x_2, x_3)$.

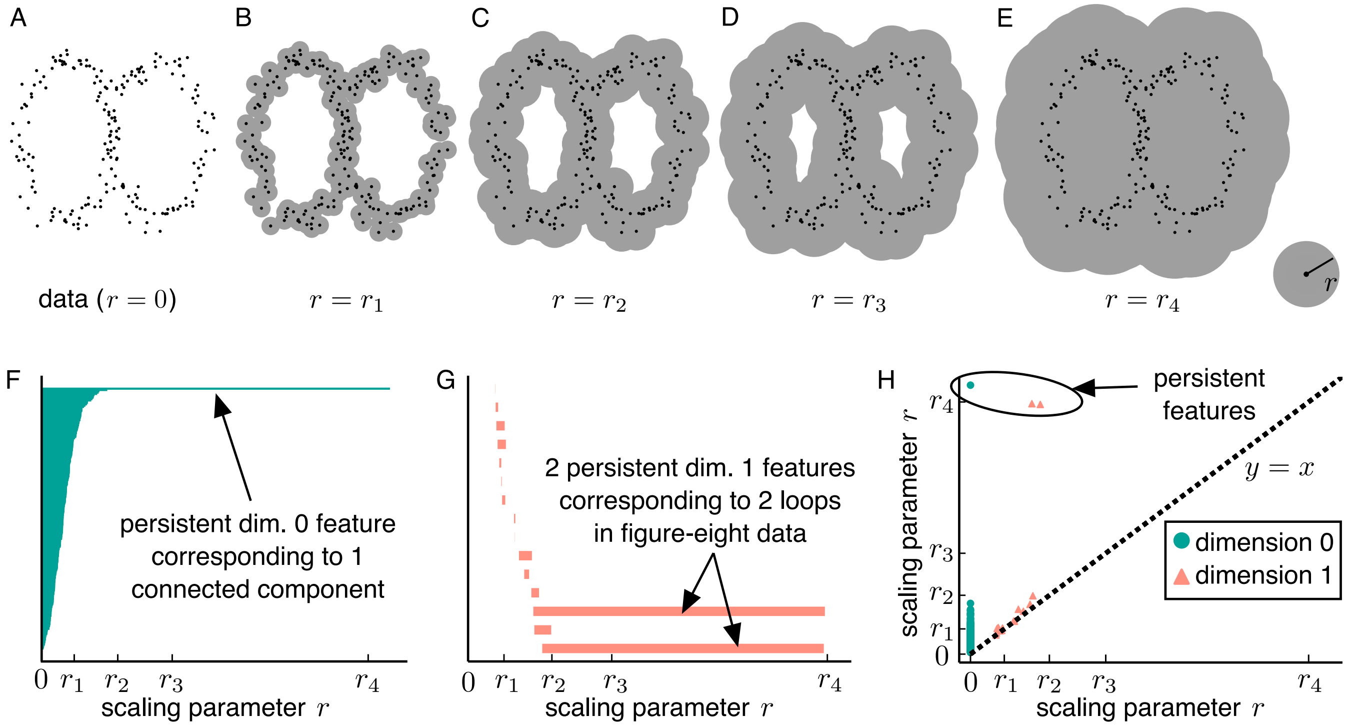

Fig. S2. Illustration of persistent homology applied to coordinate data and corresponding barcode and persistence diagrams. For easier comparison with our barcode and persistence diagrams, here we show (A) original coordinate data sampled from a figure-eight shape and (B–E) corresponding manifold expansions given by $\{b_r(\mathbf{x}_i)\}_{i=1}^N = \{\mathbf{y} \in D, i \in [1, N] : d_D(\mathbf{x}_i, \mathbf{y}) \leq r\}$ for $r = r_1 < r_2 < r_3 < r_4$ (also see Fig. 2 in the main text). (F–G) We show the dimension 0 and dimension 1 barcode diagrams that correspond to persistent homology applied to the figure-eight data. The long bar in panel (F) represents the single connected component of the figure-eight shape, and the two long bars in (G) correspond to the two loops of the figure-eight shape. (H) As an alternative means of viewing persistent homology results, we also show the analogous persistence diagram. The circular point in teal in the top left corner of the diagram represents the persistent dimension 0 feature (namely, the single connected component in our figure-eight data); and, the two triangular points in pink in the top left corner of the diagram correspond to the two persistent dimension 1 features (namely, the two loops in our figure-eight data). We calculated persistent homology using Ripser (34).

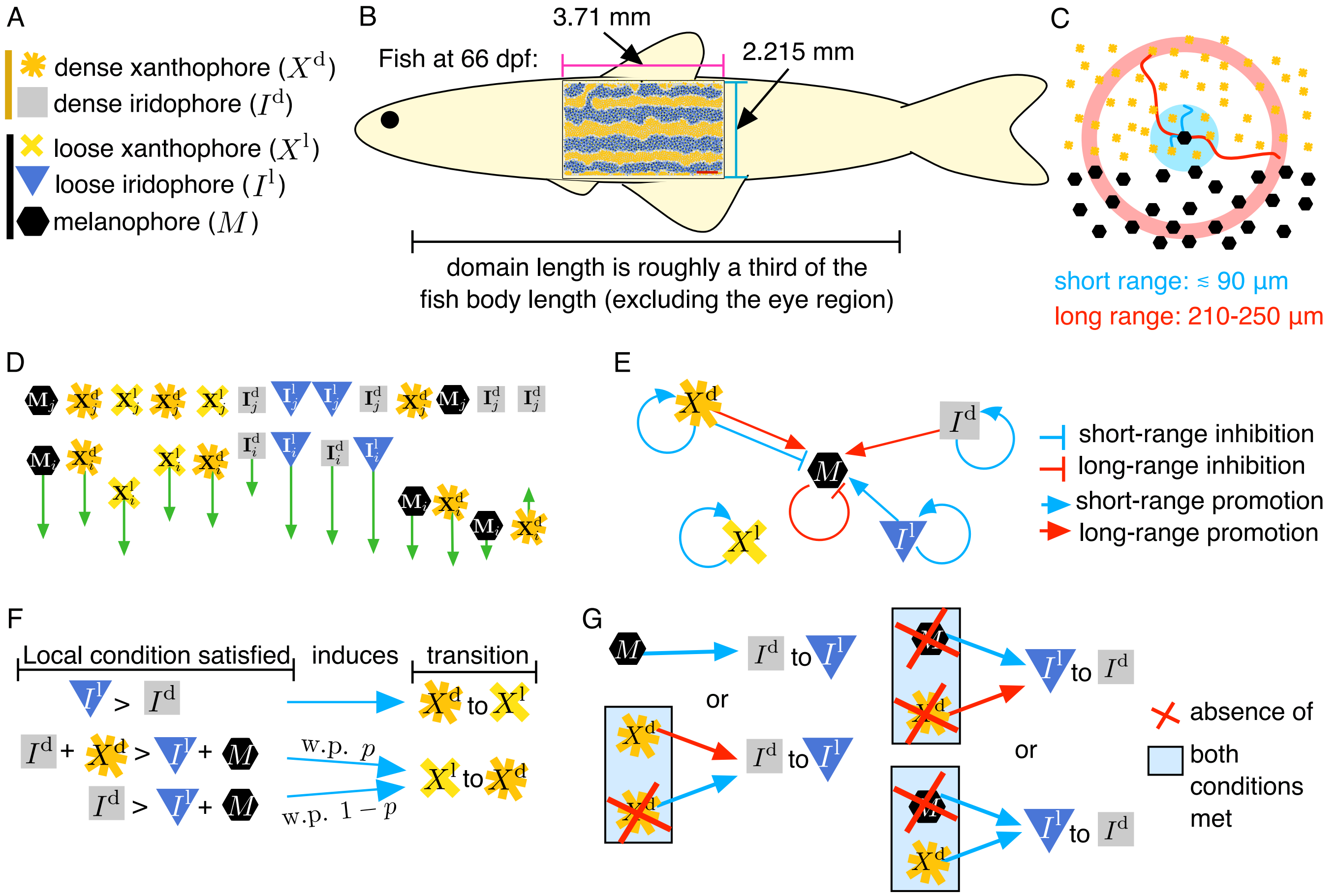

Fig. S3. Overview of the model (15) and the length scales involved. (A) The model (15) accounts for the interactions of five types of cell agents on growing rectangular domains. Each pigment cell on the fish skin is represented by an agent, and the model is parameterized to work on the same scale as cellular behavior *in vivo* (15). Reproduced from ref. (15), which is licensed under CC BY 4.0. (B) The simulation domains approximate the full fish-body height and a third of its length (except for a region around the eye) (15); the authors (15) estimated fish size with age from images in (35) to obtain the growth rates for their domains. Cartoon image is not drawn to scale. Red scale bar is $500\ \mu$m throughout this *SI Appendix*. (C) Based on measurements of cell extensions (16, 20–22), the model (15) accounts for short- and long-range interactions by considering the cells that appear in an annulus and disk around the location of interest. (D) Cell migration includes repulsion and attraction (arrow length indicates strength of force) and is described using differential equations (15). Here we show the direction that the ith cell moves in response to the jth cell. (E) We reproduce the network of short- and long-range interactions that are involved in cell differentiation and competition in the model (15). (F) Transitions in xanthophore form depend on local conditions being satisfied (15). For the cell migration, differentiation, and competition interactions in (D, E), as well as the xanthophore-form transition rules in (F), the model (15) is heavily based on known or empirically-hypothesized cell interactions. (G) In contrast, the authors (15) predicted the unknown cues that may be behind iridophore-form transitions by requiring consistency across a broad range of mutant patterns. For example, the model (15) suggests that nearby melanophores induce silver iridophores to become blue. Reproduced from ref. (15), which is licensed under CC BY 4.0.

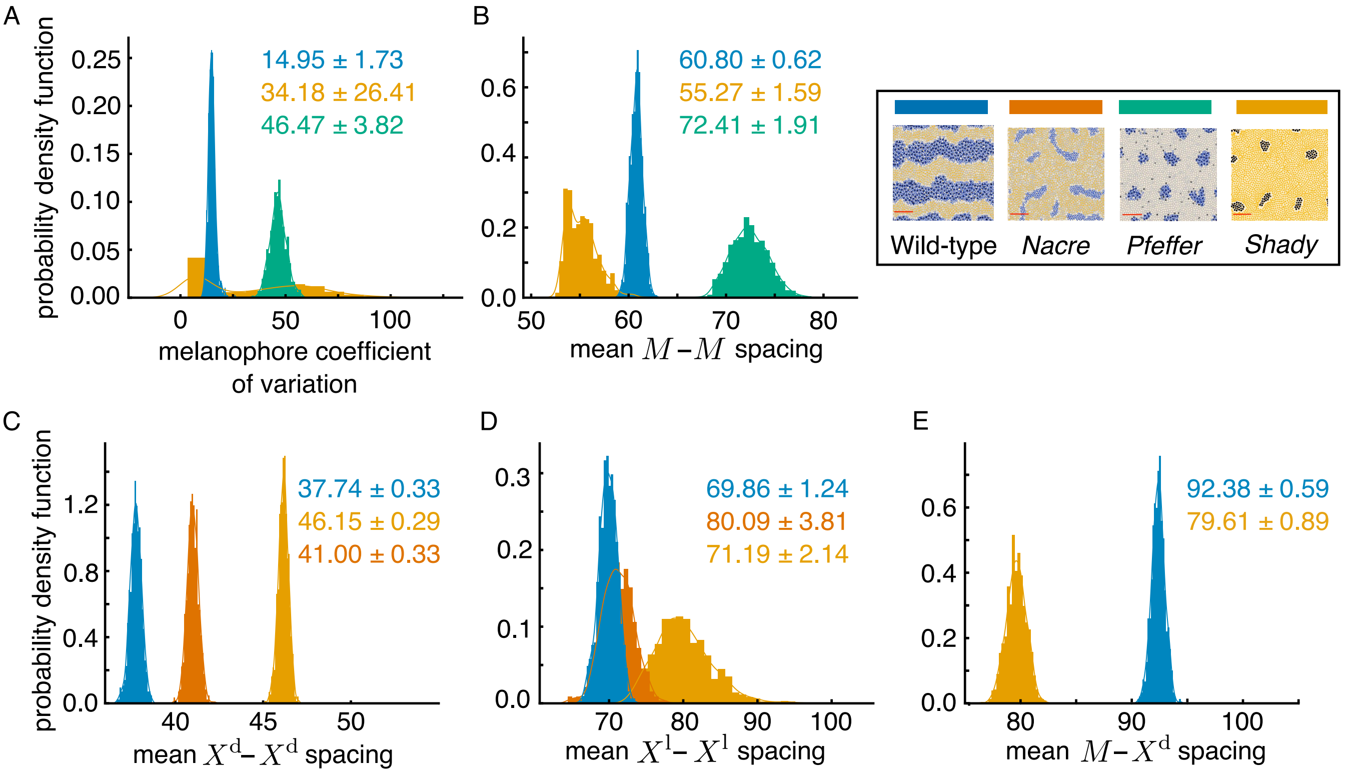

Fig. S4. Measurements of local pattern features. We show distribution plots for (A) melanophore coefficient of variation (18, 32), (B) mean M–M spacing, (C) mean X^{d}–X^{d} spacing, (D) mean X^{l}–X^{l} spacing, and (E) mean M–X^{d} spacing. We base these distributions for wild-type, *nacre*, *pfeffer*, and *shady* patterns on $1,000$ model simulations (for each pattern type) under the default parameter regime in (15). We indicate the mean plus/minus the standard deviation for these measurements in each figure.

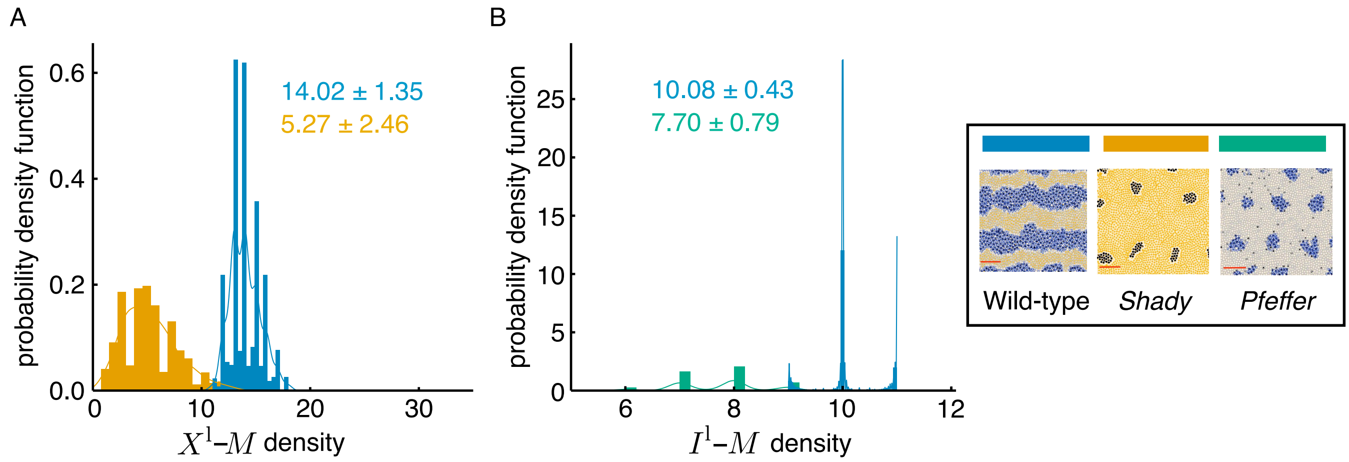

Fig. S5. Measurements of cell density. We show distribution plots for (A) X^1–M and (B) I^1–M density counts. We base these distributions for wild-type, *pfeffer*, and *shady* patterns on $1,000$ simulations of the model (15) under the default parameter regime. (We do not show distributions for *nacre* because this mutant lacks melanophores.) We indicate the mean plus/minus the standard deviation of these cell–cell density measurements in each figure.

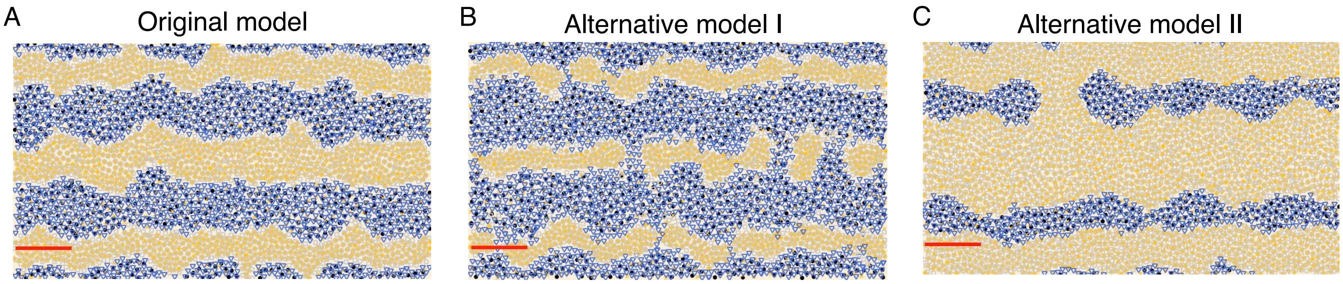

Fig. S6. Comparison of alternative models. Sample pattern simulations from (A) the original model (15) for wild-type, (B) alternative model I, and (C) alternative model II. See **Example Application: Distinguishing between Different Models** of this *SI Appendix* for more details.

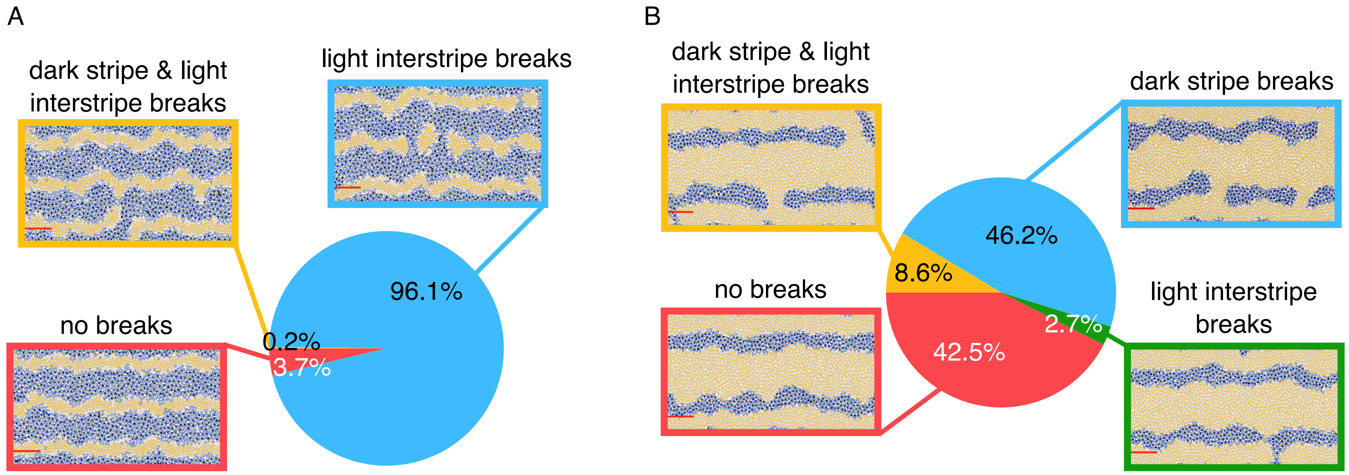

Fig. S7. Breakdown of stripe-pattern types across $1,000$ simulations of (A) alternative model I and (B) alternative model II. For each pattern type, we show a corresponding representative image. See **Example Application: Distinguishing between Different Models** of this *SI Appendix* for more details.

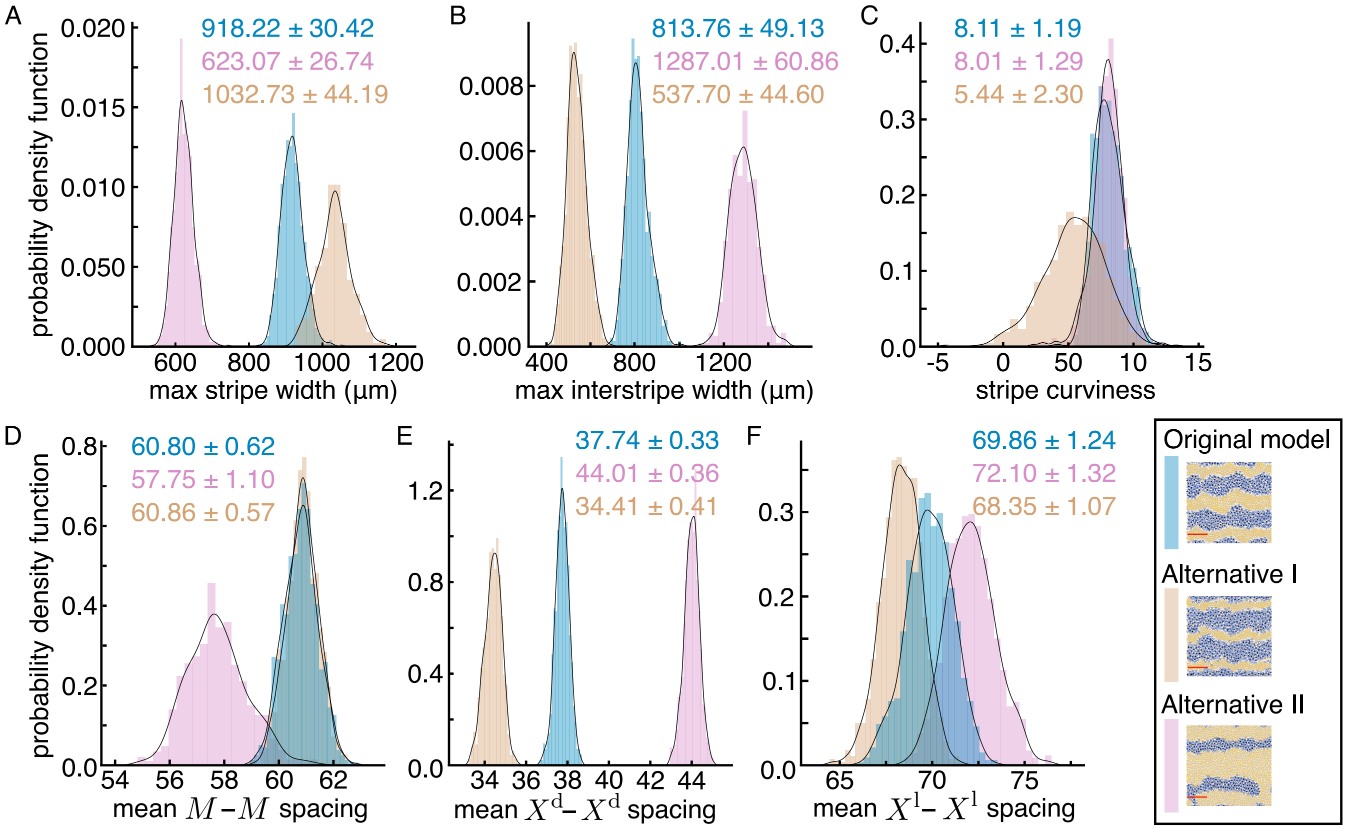

Fig. S8. Measurements of quantifiable pattern features and cell–cell distances for three different models. We show distribution plots for (A) maximum stripe separation, (B) maximum interstripe separation, (C) strive curviness, (D) mean M–M spacing, (E) mean X^{d}–X^{d} spacing, and (F) X^{l}–X^{l} spacing. We base these distributions for the original model of wild-type stripes (15), alternative model I, and alternative model II on $1,000$ simulations of each model (see **Example Application: Distinguishing between Different Models** of this *SI Appendix*). We indicate the mean plus/minus the standard deviation in each figure.

Table S1. Summary of the pattern features that we focus on and the corresponding methods that we propose for automatically quantifying these features. We define the application-specific thresholds T_p^0, T_p^1, and T_b^1 in the main text. We note that r_b is the radius value at which a given topological feature (e.g., connected component or loop) is born and r_d is the radius value at which it disappears.

**Pattern feature**	**Quantification method**
Number of spots	$\beta_0 =$ number of dimension 0 topological generators with persistence $r_d - r_b \geq T_p^0$
Number of stripes	$\beta_1 =$ number of dimension 1 topological generators with persistence $r_d - r_b \geq T_p^0$ and birth $r_b \leq T_b^1$
Stripe breaks	Check if $\beta_1 \leq$ expected number of stripes
Stripe width	$r_d - r_b$ of the dimension 1 persistence points
Spot size	Number of agents per single-linkage cluster
Stripe straightness	Arc length distances for single-linkage clusters representing stripes (see $\mathrm{Eq.}$ (2) in the main text)
Spot roundness	Ratio of PCA eigenvalues for each single-linkage cluster representing a spot (see $\mathrm{Eq.}$ (3) in the main text)
Spot alignment	Nearest-neighbor distances between single-linkage cluster centroids
Center radius	Distances from single-linkage cluster centroids to the domain midline
Time of stripe formation	Automated boundary search
Agent–agent distances	Direct calculations
Agent coefficient of variation (CV)(18, 32)	Direct calculation using $\mathrm{Eq.}$ (S16)
Agent density counts	Direct calculation of local counts

Table S2. Quantifying variability and breakdown for wild-type zebrafish patterns as a function of additional stochasticity in cell interactions (also see Fig. 6 in the main text). We base these measurements on $1,000$ simulations (for each value of σ) of our adapted version of the model (15), adjusted as described in the text of this *SI Appendix*. We choose cell-interaction length-scale parameters randomly each day per cell and interaction from a normal distribution (centered at the default length scale) with increasing standard deviation given by σ times the default length scale.

	less stochasticity ←				→ more stochasticity	
**Noise strength σ**	**0.01**	**0.05**	**0.1**	**0.2**	**0.3**	**0.5**
Percent of patterns with no breaks	80.70%	82.70%	80.40%	72.50%	64.90%	24.80%
Percent of patterns with stripe breaks only	3.90%	3.50%	4.40%	5.60%	8.30%	45.90%
Percent of patterns with interstripe breaks only	13.70%	11.80%	11.90%	18.60%	20.80%	9.60%
Percent of patterns with stripe & interstripe breaks	1.70%	2.00%	3.30%	3.30%	6.00%	19.70%
Mean maximum stripe width (μm)	899.74	892.82	895.82	911.58	932.44	894.04
Standard deviation in maximum stripe width (μm)	27.64	29.45	29.54	31.33	34.80	46.50
Mean maximum interstripe width (μm)	766.02	780.12	792.54	820.20	844.60	903.34
Standard deviation in maximum interstripe width (μm)	45.88	44.82	43.87	46.34	56.35	83.07
Mean stripe curviness measure	7.21%	7.28%	7.50%	8.41%	9.87%	11.59%
Standard deviation in stripe curviness	1.32%	1.24%	1.22%	1.21%	1.36%	4.27%
Mean time of interstripe X1D & X1V formation (dpf)	41.14	40.21	40.40	40.64	41.11	42.29
Standard deviation in X1D & X1V formation (dpf)	1.26	1.24	1.26	1.29	1.31	1.55

Table S3. Quantifying pattern variability and breakdown for the *nacre* mutant as a function of additional stochasticity in cell interactions (also see Fig. 6 in the main text). We base these measurements on $1,000$ simulations (for each value of σ) of our adapted version of the model (15), adjusted as described in the text of this *SI Appendix*. We choose cell-interaction length-scale parameters each day randomly per cell and interaction from a normal distribution (centered at the default length scale) with increasing standard deviation given by σ times the default length scale.

	less stochasticity ←				→ more stochasticity
**Noise strength σ**	**0.01**	**0.05**	**0.1**	**0.2**	**0.3**	**0.5**
Percent of patterns with normal spots	99.40%	98.80%	97.50%	45.50%	0.00%	0.00%
Percent of patterns with small spots	0.10%	0.00%	1.40%	54.30%	27.00%	0.00%
Percent of patterns with many spots	0.50%	1.20%	1.00%	0.00%	0.00%	0.00%
Percent of patterns with small spots and many spots	0.00%	0.00%	0.10%	0.20%	73.0%	100.0%
Mean spot roundness score	6.45	6.07	5.81	4.81	4.22	10.59
Standard deviation in spot roundness score	6.17	6.03	5.63	4.31	3.26	20.23
Mean variance in spot spacing (μm)	337.17	339.36	344.00	375.56	388.81	148.18
Standard deviation in variance in spot spacing (μm)	67.07	72.56	75.74	108.11	143.95	64.99
Mean X0 interstripe width (μm)	1044.92	1064.99	1073.66	1338.23	2860.02	2001.47
Standard deviation in X0 interstripe width (μm)	416.42	448.59	441.43	672.62	973.30	1171.27

Table S4. Quantifying pattern variability and breakdown for the *pfeffer* mutant as a function of additional stochasticity in cell interactions (also see Fig. 6 in the main text). We base these measurements on $1,000$ simulations (for each value of σ) of our adapted version of the model (15), adjusted as described in the text of this *SI Appendix*. We choose cell-interaction length-scale parameters each day randomly per cell and interaction from a normal distribution (centered at the default length scale) with increasing standard deviation given by σ times the default length scale.

	← less stochasticity					more stochasticity →
**Noise strength σ**	**0.01**	**0.05**	**0.1**	**0.2**	**0.3**	**0.5**
Percent of patterns with normal spots	82.30%	88.40%	83.50%	15.70%	0.10%	0.00%
Percent of patterns with small spots	17.70%	11.40%	16.30%	75.80%	16.80%	0.70%
Percent of patterns with few spots	0.00%	0.10%	0.20%	0.00%	0.00%	0.00%
Percent of patterns with small spots and many spots	0.00%	0.10%	0.00%	8.50%	83.10%	99.30%
Mean spot roundness score	2.32	2.30	2.29	2.42	2.76	4.97
Standard deviation in spot roundness score	0.67	0.62	0.64	0.76	1.14	7.68
Mean variance in spot spacing (μm)	330.29	333.95	337.69	325.61	290.93	286.42
Standard deviation in variance in spot spacing (μm)	30.23	30.18	31.14	37.26	50.14	101.07
Mean X0 interstripe width (μm)	920.98	919.72	936.03	1000.12	1132.60	2304.91
Standard deviation in X0 interstripe width (μm)	155.33	140.40	150.40	187.53	386.15	1040.17

Table S5. Quantifying pattern variability and breakdown for the *shady* mutant as a function of additional stochasticity in cell interactions (also see Fig. 6 in the main text). We base these measurements on $1,000$ simulations (for each value of σ) of our adapted version of the model (15), adjusted as described in the text of this *SI Appendix*. We choose cell-interaction length-scale parameters each day randomly per cell and interaction from a normal distribution (centered at the default length scale) with increasing standard deviation given by σ times the default length scale.

	← less stochasticity				more stochasticity →	
**Noise strength σ**	**0.01**	**0.05**	**0.1**	**0.2**	**0.3**	**0.5**
Percent of patterns with normal spots	100.00%	99.30%	97.30%	47.80%	1.40%	0.00%
Percent of patterns with small spots	0.00%	0.20%	0.00%	0.00%	0.30%	0.00%
Percent of patterns with few spots	0.00%	0.40%	2.70%	52.20%	86.00%	12.60%
Percent of patterns with no spots	0.00%	0.00%	0.00%	0.00%	0.10%	70.70%
Percent of patterns with small spots and few spots	0.00%	0.00%	0.00%	0.00%	11.90%	16.60%
Percent of patterns with big spots and few spots	0.00%	0.10%	0.00%	0.00%	0.30%	0.10%
Mean roundness score	2.85	2.68	2.43	2.13	2.55	2.53
Standard deviation in spot roundness score	1.08	0.98	0.86	0.86	2.14	1.22
Mean variance in spot spacing (μm)	455.06	462.97	472.76	537.76	789.41	N/A
Standard deviation in variance in spot spacing (μm)	50.02	53.29	56.17	96.30	279.46	N/A
Mean X0 interstripe width (μm)	1239.53	1239.42	1247.12	1366.71	2044.69	4278.00
Standard deviation in X0 interstripe width (μm)	188.39	191.90	192.84	262.18	820.31	0.00

Table S6. Results from two-sided Kolmogorov–Smirnov tests for the null hypothesis that two independent samples are drawn from the same continuous distribution. Here we compare the distributions of pattern features from the original model for wild-type stripe formation (15), alternative (alt.) model I, and alternative model II (see Example Application: Distinguishing between Different Models of this *SI Appendix*). For each test we show the Kolmogorov–Smirnov test statistic and corresponding p-value. A small p-value (namely, $p < 0.01$) indicates that we can reject the null hypothesis that the two independent samples are drawn from the same continuous distribution. We highlight non-significant test results with italic, bold font. We performed the Kolmogorov–Smirnov in Python 3.0 using Scipy.

Pattern feature	Original model vs. alt. model I	Original model vs. alt. model II	Alt. model I vs. alt. model II
Maximum stripe width	$p = 0.0$, KS statistic $= 0.938$	$p = 0.0$, KS statistic $= 0.995$	$p = 0.0$, KS statistic $= 0.995$
Maximum interstripe width	$p = 0.0$, KS statistic $= 0.990$	$p = 0.0$, KS statistic $= 0.904$	$p = 0.0$, KS statistic $= 0.991$
Stripe curviness	$p = 3.6e^{-161}$, KS statistic $= 0.605$	***p = 0.094, KS statistic = 0.055***	$p = 5.4e^{-158}$, KS statistic $= 0.599$
Mean M–M spacing	***p = 0.016, KS statistic = 0.069***	$p = 0.0$, KS statistic $= 0.924$	$p = 0.0$, KS statistic $= 0.934$
Mean X^{d}–X^{d} spacing	$p = 0.0$, KS statistic $= 1.0$	$p = 0.0$, KS statistic $= 1.0$	$p = 0.0$, KS statistic $= 1.0$
Mean X^{l}–X^{l} spacing	$p = 2.0e^{-112}$, KS statistic $= 0.505$	$p = 7.8e^{-164}$, KS statistic $= 0.610$	$p = 0.0$, KS statistic $= 0.893$

References

1. Carlsson G (2009) Topology and data. *Bull. Am. Math. Soc.* 46(2):255–308.
2. Chazal F, de Silva V, Glisse M, Oudot S (2016) *The Structure and Stability of Persistence Modules.* (Springer International Publishing), 1.0 edition.
3. Edelsbrunner H, Harer JL (2010) *Computational Topology, An Introduction.* (American Mathematical Society).
4. Ghrist R (2014) *Elementary Applied Topology.* (Createspace), 1.0 edition.
5. Zomorodian A (2009) *Topology for Computing.* (Cambridge University Press).
6. Curto C (2017) What can topology tell us about the neural code? *Bull. Am. Math. Soc.* 54(1):63–78.
7. Giusti C, Pastalkova E, Curto C, Itskov V (2015) Clique topology reveals intrinsic geometric structure in neural correlations. *Proc. Natl. Acad. Sci. U.S.A.* 112(44):13455–13460.
8. Sizemore AE, et al. (2018) Cliques and cavities in the human connectome. *J. Comput. Neurosci.* 44(1):115–145.
9. Chan JM, Carlsson G, Rabadan R (year?).
10. Camara PG, Rosenbloom DI, Emmett KJ, Levine AJ, Rabadan R (2016) Topological data analysis generates high-resolution, genome-wide maps of human recombination. *Cell Systems* 3(1):83 – 94.
11. Humphreys DP, McGuirl MR, Miyagi M, Blumberg AJ (2019) Fast estimation of recombination rates using topological data analysis. *Genetics* 211:1–14.
12. Munch E, Shapiro M, Harer J (2012) Failure filtrations for fenced sensor networks. *Int. J. Robotics Res.* 31(9):1044–1056.
13. Adams H, Carlsson G (2015) Evasion paths in mobile sensor networks. *Int. J. Robotics Res.* 34(1):90–104.
14. Hatcher A (2000) *Algebraic topology.* (Cambridge University Press, Cambridge).
15. Volkening A, Sandstede B (2018) Iridophores as a source of robustness in zebrafish stripes and variability in *Danio* patterns. *Nat. Commun.* 9(3231).
16. Mahalwar P, Singh AP, Fadeev A, Nüsslein-Volhard C, Irion U (2016) Heterotypic interactions regulate cell shape and density during color pattern formation in zebrafish. *Biol. Open* 5(11):1680–1690.
17. Takahashi G, Kondo S (2008) Melanophores in the stripes of adult zebrafish do not have the nature to gather, but disperse when they have the space to move. *Pigment Cell Melanoma Res.* 21(6):677–686.
18. Parichy DM, Turner JM (2003) Zebrafish *puma* mutant decouples pigment pattern and somatic metamorphosis. *Dev. Biol.* 256(2):242–257.
19. Yamanaka H, Kondo S (2014) In vitro analysis suggests that difference in cell movement during direct interaction can generate various pigment patterns in vivo. *Proc. Natl. Acad. Sci. U.S.A.* 111(5):1867–1872.
20. Inaba M, Yamanaka H, Kondo S (2012) Pigment pattern formation by contact-dependent depolarization. *Science* 335(6069):677–677.
21. Hamada H, et al. (2014) Involvement of delta/notch signaling in zebrafish adult pigment stripe patterning. *Development* 141(2):318–324.
22. Eom DS, Bain EJ, Patterson LB, Grout ME, Parichy DM (2015) Long-distance communication by specialized cellular projections during pigment pattern development and evolution. *eLife* 4:e12401.
23. Budi EH, Patterson LB, Parichy DM (2011) Post-embryonic nerve-associated precursors to adult pigment cells: genetic requirements and dynamics of morphogenesis and differentiation. *PLoS Genet.* 7(5):e1002044.
24. Dooley CM, Mongera A, Walderich B, Nüsslein-Volhard C (2013) On the embryonic origin of adult melanophores: the role of Erbb and Kit signalling in establishing melanophore stem cells in zebrafish. *Development* 140(5):1003–1013.
25. Mahalwar P, Walderich B, Singh AP, Nüsslein-Volhard C (2014) Local reorganization of xanthophores fine-tunes and colors the striped pattern of zebrafish. *Science* 345(6202):1362–1364.
26. Patterson LB, Parichy DM (2013) Interactions with iridophores and the tissue environment required for patterning melanophores and xanthophores during zebrafish adult pigment stripe formation. *PLOS Genet.* 9(5).
27. Nakamasu A, Takahashi G, Kanbe A, Kondo S (2009) Interactions between zebrafish pigment cells responsible for the generation of Turing patterns. *Proc. Natl. Acad. Sci. U.S.A.* 106(21):8429–8434.
28. Singh AP, Schach U, Nüsslein-Volhard C (2014) Proliferation, dispersal and patterned aggregation of iridophores in the skin prefigure striped colouration of zebrafish. *Nat. Cell Biol.* 16(6):604–611.
29. McMenamin SK, et al. (2014) Thyroid hormone-dependent adult pigment cell lineage and pattern in zebrafish. *Science* 345(6202):1358–1361.
30. Yamaguchi M, Yoshimoto E, Kondo S (2007) Pattern regulation in the stripe of zebrafish suggests an underlying dynamic and autonomous mechanism. *Proc. Natl. Acad. Sci. U.S.A.* 104(12):4790–4793.
31. Patterson LB, Bain EJ, Parichy DM (2014) Pigment cell interactions and differential xanthophore recruitment underlying zebrafish stripe reiteration and *Danio* pattern evolution. *Nat. Commun.* 5.
32. Parichy DM, Turner JM (2003) Temporal and cellular requirements for fms signaling during zebrafish adult pigment pattern development. *Development* 130(5):817–833.
33. Frohnhöfer HG, et al. (2016) Spermidine, but not spermine, is essential for pigment pattern formation in zebrafish. *Biol. Open* 5(6):736–744.
34. Bauer U (2019) Ripser: Efficient computation of Vietoris–Rips persistence barcodes. 1908.02518.
35. Parichy DM, Elizondo MR, Mills MG, Gordon TN, Engeszer RE (2009) Normal table of postembryonic zebrafish development: staging by externally visible anatomy of the living fish. *Dev. Dyn.* 238(12):2975–3015.
